# Colchicine-mediated selective autophagic degradation of HBV core proteins inhibits HBV replication and HBV-related hepatocellular carcinoma progression

**DOI:** 10.1038/s41420-024-02122-z

**Published:** 2024-08-06

**Authors:** Hui Zhang, Xiameng Su, Leirong Gu, Ming Tan, Yuting Liu, Kexin Xu, Jihua Ren, Juan Chen, Zhihong Li, Shengtao Cheng

**Affiliations:** 1 Department of Clinical Laboratory, The Second Affiliated Hospital of Chongqing Medical University, Chongqing, China; 2 The Key Laboratory of Molecular Biology of Infectious Diseases Designated by the Chinese Ministry of Education, Chongqing Medical University, Chongqing, China; 3 The State Key Laboratory of Ultrasound in Medicine and Engineering, College of Biomedical Engineering, Chongqing Medical University, Chongqing, 400016 P. R. China

**Keywords:** Viral hepatitis, Tumour virus infections

## Abstract

The HBV core protein (HBc) is an important viral protein of HBV that plays an indispensable role in the lifecycle of HBV, including capsid assembly and transport, reverse transcription and virus release. In recent years, evidence has shown that HBc may be involved in the malignant progression of HCC. Thus, HBc is an attractive target for antiviral agents and provides a new strategy for the treatment of HBV-related HCC. Here, we identified a novel anti‐HBc compound—colchicine, an alkaloid compound—that promoted selective autophagic degradation of HBc through the AMPK/mTOR/ULK1 signalling pathway. We further confirmed that colchicine promoted the selective autophagy of HBc by enhancing the binding of HBc to the autophagy receptor p62. Finally, we evaluated the effects of colchicine on HBV replication and HBc-mediated HCC metastasis in vitro and in vivo. Our research indicated that the inhibitory effects of colchicine on HBV and HBV-related HCC depend on the selective autophagic degradation of HBc. Thus, colchicine is not only a promising therapeutic strategy for chronic hepatitis B but also a new treatment for HBV-related HCC.

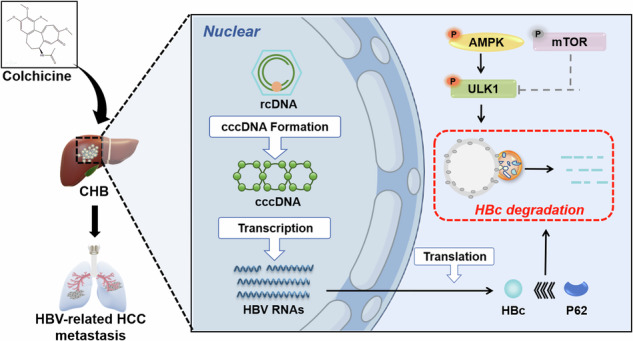

## Introduction

Hepatitis B virus (HBV) infection is a global health problem that cannot be ignored [1] and contributes to more than half of hepatocellular carcinoma (HCC) cases worldwide [2, 3]. Although effective preventive vaccines have become available in recent decades, chronic HBV infection remains difficult to cure. Current drugs for chronic HBV infection include IFN-α, its pegylated form and long-term nucleos(t)ide analogues (NUCs). Both approaches result in very low rates of HBsAg clearance [4]. There is a great medical need to develop new drugs to improve the CHB cure rate.

Hepatitis B virus (HBV) contains 3.2 kb of DNA and is a hepatotropic DNA virus. The HBV genome is composed of double-stranded incomplete circular DNA, which can form covalently closed circular DNA (cccDNA) in the nucleus of infected cells and can be further transcribed and translated to produce viral proteins, including viral polymerase (HBp), X protein (HBx), envelope proteins (HBs), HBeAg, and core/capsid proteins (HBc). Targeting viral proteins to cure hepatitis B has become a research hotspot [5–7], and the development of HBc-targeting compounds has become a potential direction.

HBc is a 21 kDa protein involved in the formation of the viral nucleocapsid and plays an important role in viral capsid assembly and transport, reverse transcription and viral release. [8] Recently, it has been reported that allosteric regulation of core proteins can inhibit HBV replication and the formation of new cccDNA [9, 10]. However, the role of HBc is not limited to HBV. In recent years, it has been found that HBc plays an important role in the malignant transformation of HCC [11], and HBc has been found to be involved in the activation of cell signalling pathways related to cell proliferation and migration [12–14]. Therefore, HBc is an attractive target for inhibiting HBV and HBV-related HCC.

Alkaloids are organic compounds with a cyclic ring structure containing one or more basic nitrogen atoms [15] and are often used to treat infectious diseases [16] and antiviral agents [17]. In this study, we identified a novel anti‐HBc compound, colchicine, by screening an alkaloid compound library with a novel screening strategy. Moreover, we elucidated the molecular mechanism by which colchicine (Col) promotes the binding of the cargo receptor p62 to HBc and induces selective autophagic degradation of HBc. Given the dual role of HBc in HBV replication and HCC metastasis, our study provides deeper insight into the mechanism by which colchicine inhibits HBV replication and HBV-related HCC metastasis.

## Results

### Alkaloid compound screening for compounds targeting HBc

**Fig. 1:**
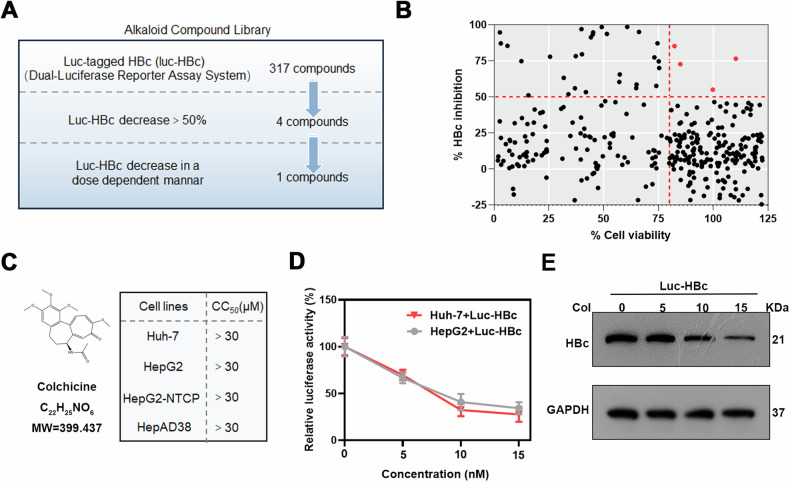
Identification of anti‐HBc compounds. **A** Schematic diagram of the screening of anti-HBc alkaloid compounds. **B** Evaluation of the HBc inhibition rates and cell viability of the 317 alkaloid compounds. **C** Chemical structure of colchicine and cytotoxicity of colchicine in four cell lines. **D**, **E** After the transfection of Huh-7 and HepG2 cells with Luc-HBc for 24 h, Col treatment was performed for 48 h. **D** The relative luciferase activity of HBc were examined by dual luciferase reporter assay in Huh-7 and HepG2 cells. **E** The protein level of Luc-HBc in Huh-7 cells was examined by western blotting. The data was expressed as the mean ± SD of three replicates of the experiment.

To screen for alkaloid compounds that inhibit HBc, a compound library containing 317 alkaloid compounds was used for screening (Fig. 1A). The activity of HBc was determined by transfection of HBc luciferase reporter plasmid (Luc-HBc) and dual luciferase reporter assay (Fig. S1A). To detect the inhibitory and drug toxicity of the compounds, Huh-7 cells expressing Luc-HBc were exposed to alkaloid compounds for 2 days, and luciferase activity and cell viability were detected to evaluate the effects of the compounds. The four compounds reduced the Luc-HBc level by 50% or more, and no significant cytotoxicity was observed (Fig. 1B). Further study revealed that only colchicine effectively inhibited Luc-HBc expression in a dose-dependent manner (Fig. 1D, E and S1B-E). The chemical structure and cytotoxicity of colchicine was shown in Fig. 1C. We found that the CC_50_ of colchicine was greater than 30 μM in the Huh-7, HepG2, HepG2-NTCP and HepAD38 cell lines, and no significant cytotoxicity was observed in these cells at the concentrations used (maximum concentration used: 15 nM) (Fig. 1C and S2A–D). Finally, we chose colchicine for further study.

### Colchicine induces HBc degradation through the autophagy pathway

**Fig. 2:**
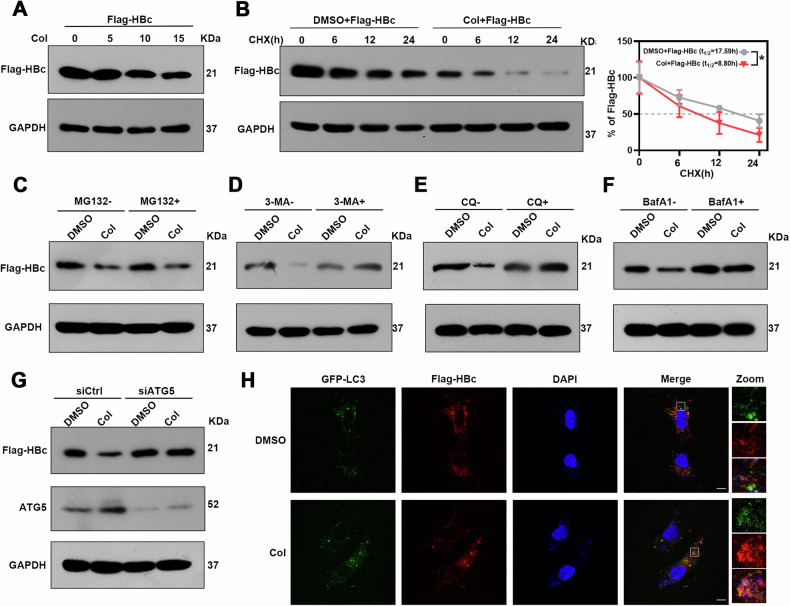
Colchicine promotes the autophagic degradation of HBc. **A**–**G** Huh-7 cells were transfected with plasmids encoding Flag-HBc. **A** Cells were treated with Col for 48 h. The HBc protein level was determined via western blotting. **B** Cells were treated with colchicine or DMSO for 48 h and then subjected to 10 μg/ml cycloheximide, and determine HBc protein levels using western blotting. The half-life of HBc was calculated by three independent experiments. (*P < 0.05). **C**–**F** Cells were treated with or without Col for 24 h post-transfection, and then the cells were treated with or without MG132 (10 μM), 3-MA (500 μM), CQ (50 μM), or BafA1 (0.1 μM) for 24 h. Determine HBc protein levels using western blotting. **G** Huh-7 cells were transduced with siCtrl or siATG5, transfected with a plasmid encoding Flag-HBc, and then treated with or without Col for 48 h. Determine Flag-HBc and ATG5 protein levels using western blotting. **H** Huh-7 cells were transfected with a plasmid encoding GFP-LC3, and Flag-HBc, GFP-LC3 and Flag-HBc were visualized by immunofluorescence staining. Scale bar = 10 μm. The data was expressed as mean ± SD of three replicates of the experiment.

To further explore the effect of colchicine on HBc, we transfected Huh-7 cells with a plasmid encoding Flag-HBc, and western blotting and real-time PCR were used to evaluate the effects of colchicine on HBc protein and mRNA levels. We found that colchicine treatment reduced HBc protein levels in a dose-dependent manner but had no effect on the HBc mRNA level (Fig. 2A and S3A and C). Meanwhile, when studying the effect of colchicine on HBc at different treatment times, we found no difference between 48 and 72 h of colchicine treatment (Fig. S3B). Therefore, we decided to continue using 48 h as the treatment time for colchicine in subsequent experiments. Further research found that there were no significant differences in the protein or mRNA levels of Flag-HBx, Flag-HBs or Flag-HBp after colchicine treatment (Fig. S3A and S3D–F). This finding suggested that colchicine specifically degrades HBc proteins. Using the cycloheximide (CHX) chase method, we next sought to determine whether the half-life of the HBc protein was affected by colchicine and found that colchicine treatment significantly shortened the half-life of the HBc protein (Fig. 2B). We further explored the mechanism of HBc degradation and found that the colchicine‐induced reduction in HBc expression was markedly blocked by the autophagy inhibitors 3-MA, CQ, and especially BafA1 but not by MG132, which is a proteasome inhibitor (Fig. 2C–F). Consistently, silencing ATG5, an essential molecule for autophagy, also counteracted the inhibitory effect of colchicine on HBc levels (Fig. 2G). Further analysis by confocal microscopy revealed the colocalization of HBc and LC3 in Col-treated cells (Fig. 2H). Thus, colchicine may inhibit HBc expression by promoting autophagic degradation of the viral protein HBc.

### p62 is involved in the colchicine-induced autophagic degradation of HBc

**Fig. 3:**
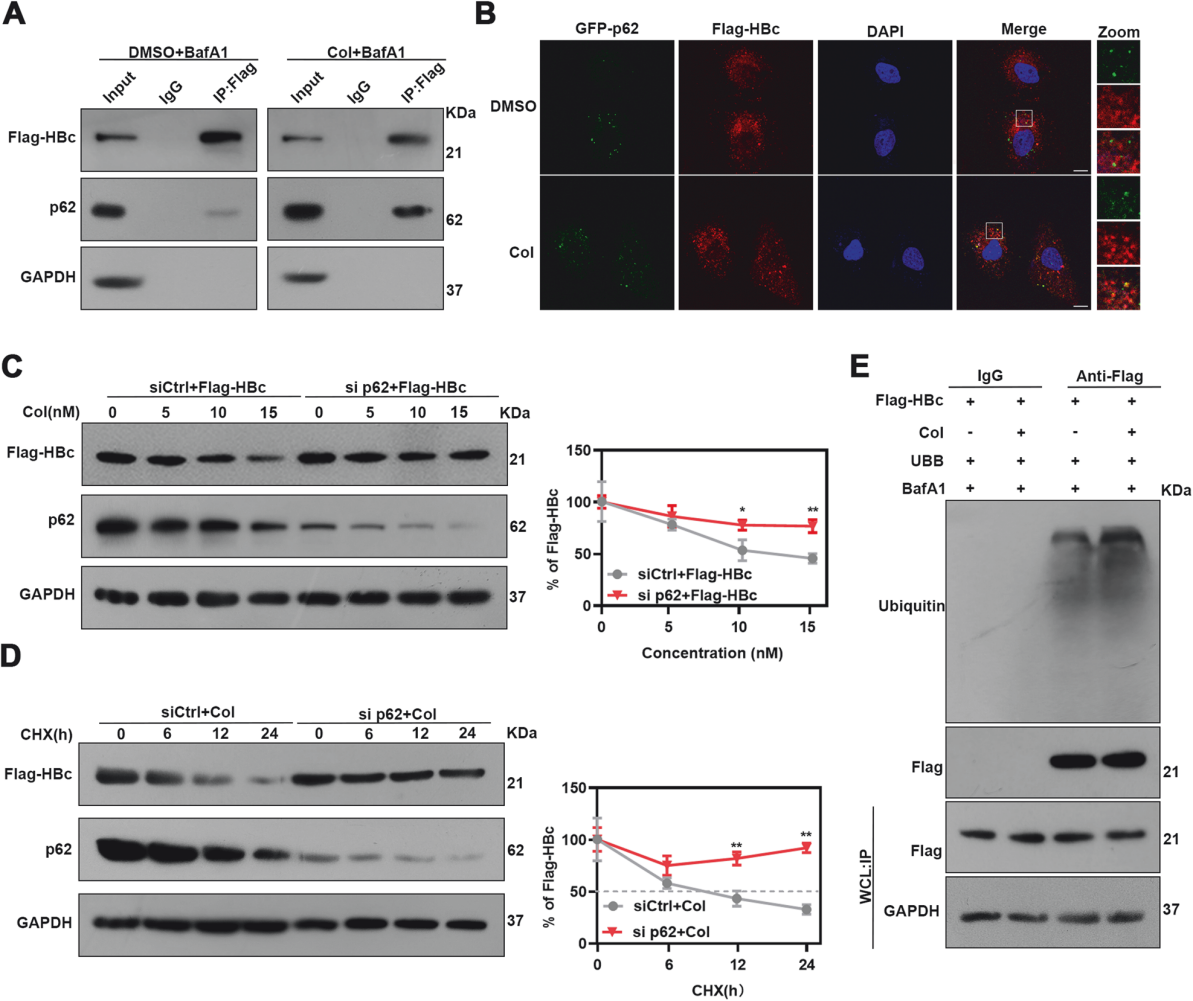
Colchicine degrades HBc via p62-mediated selective autophagy. **A** Transfect plasmids encoding Flag-HBc and GFP-p62 into Huh-7 cells, and then treat with or without Col for for 24 h, and then treated with BafA1 for 24 h. Using anti-Flag antibodies to precipitate cell lysate and then perform immunoblotting. **B** Immunofluorescence staining was used to detect the colocalization of GFP-p62 and Flag-HBc in Huh-7 cells. Scale bar = 10 μm. **C**, **D** Huh-7 cells were transduced with siCtrl or si p62 and then transfected with a plasmid encoding Flag-HBc. **C** Treat cells with different concentrations of Col for 48 h. The protein level of Flag-HBc and p62 was examined by western blotting. **D** Treat cells with Col for 48 h and then subjected to CHX (10 μg/ml). The protein level of Flag-HBc and p62 was examined by western blotting. **E** Transfect plasmids encoding Flag-HBc and HA-Ub into Huh-7 cells, treated with Col for 24 h, and then treated with BafA1 for 24 h. Immunoprecipitation of whole cell extract with anti-Flag antibody and detection of ubiquitinated HBc with anti-ubiquitin antibody.

p62, a classic autophagy receptor, is a multifunctional protein located throughout the cell that is involved in the degradation of many exogenous proteins, including viral proteins. HBc reportedly interacts with p62. Hence, we first examined the interaction of HBc with p62 [18]. Co-IP analysis revealed that HBc could bind to p62, and colchicine treatment promoted the interaction between HBc and p62 (Fig. 3A). Consistently, confocal microscopy revealed obvious colocalization of p62 and HBc in Col-treated cells (Fig. 3B). However, the GST pull-down analysis indicated that direct binding of p62 to HBc was unlikely (Fig. S4A) and silencing p62 had no significant effect on HBc levels (Fig. S4B), whereas silencing p62 in colchicine-treated cells interrupted the inhibitory effect of colchicine on HBc (Fig. 3C). Additionally, a protein CHX chase assay showed that p62 depletion interfered with Col-induced HBc degradation (Fig. 3D). These data suggest that p62 is involved in the degradation of HBc mediated by colchicine. Because HBc ubiquitination is an essential step in p62-mediated HBc degradation. Therefore, the effect of colchicine on HBc ubiquitination was tested. We found that HBc was highly ubiquitinated after treatment with colchicine (Fig. 3E). Taken together, these data indicated that colchicine enhances the p62-mediated ubiquitination and degradation of HBc.

### Colchicine induces autophagy through the AMPK/mTOR/ULK1 signalling pathway

**Fig. 4:**
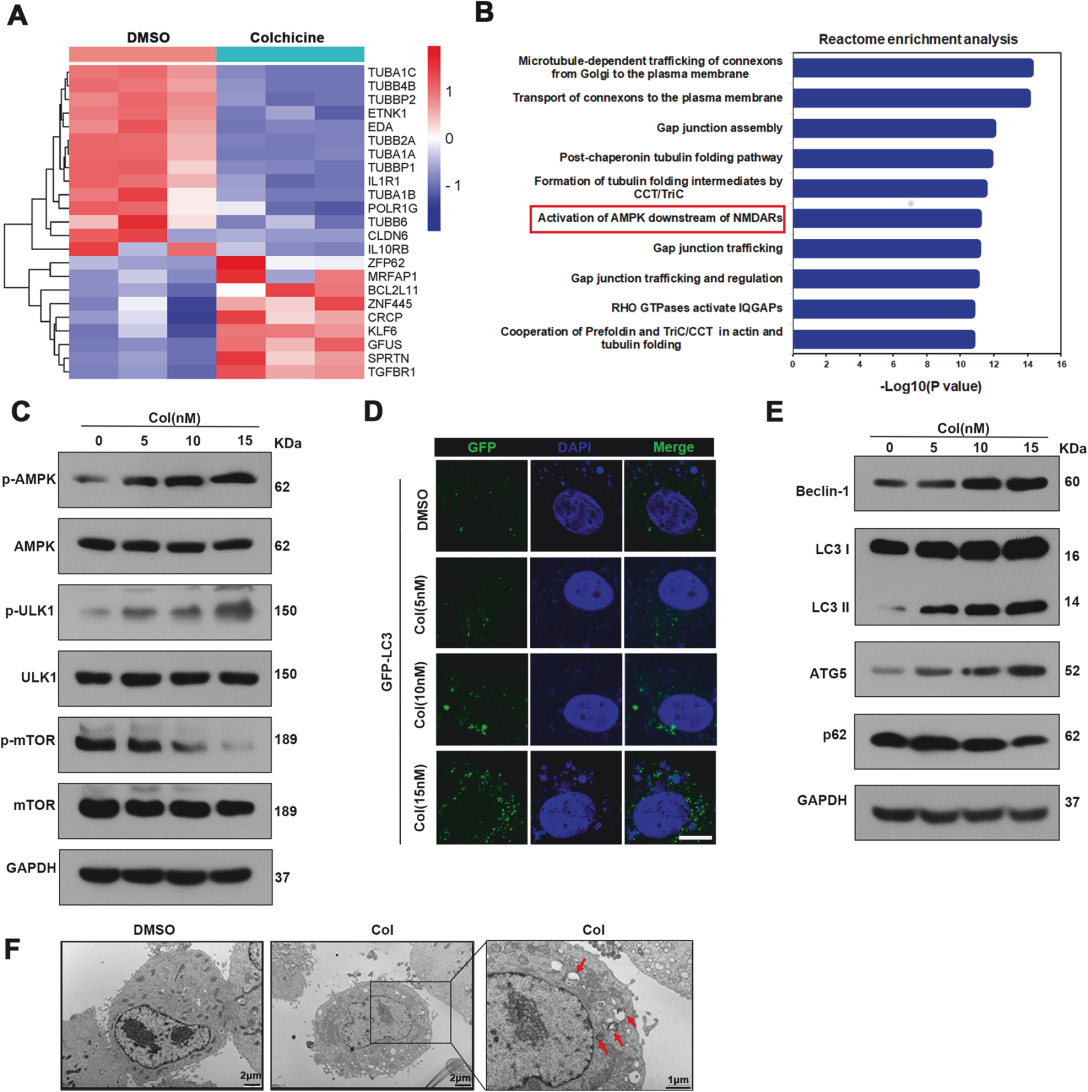
Colchicine induced autophagic degradation via the AMPK/mTOR/ULK1 signalling pathway. **A** HepG2-NTCP cells infected with HBV were treated with or without Col for 2 days. Total RNA was isolated for RNA-seq. A heatmap of the significantly differentially expressed mRNAs was constructed. Grey indicates no change, bright blue indicates decreased expression, and bright red indicates overexpression. (DMSO, *n* = 3; Col, *n* = 3) ( | log_2_FC | >1, *p*-value < 0.05). **B** Reactome enrichment analysis of the DEGs. The top 10 items are presented according to the *P* value. **C** Transfect plasmids encoding Flag-HBc into Huh-7 cells, and then treated with Col for 48 h. The protein levels of AMPK, ULK1, and mTOR and their corresponding phosphorylated forms were measured by western blotting. **D** Transfect plasmids encoding GFP-LC3 into Huh-7 cells, and then treated with Col for 48 h. The level of LC3 was measured by immunofluorescence. Scale bar = 10 μm. **E**, **F** Transfect plasmids encoding Flag-HBc into Huh-7 cells, and then treated with Col for 48 h. **E** The protein levels of Beclin1 and ATG5, the LC3B-II/I ratio and the expression level of p62 were measured by western blotting. **F** Electron microscopy was used to observe autophagolysosomes (red squares).

To elucidate the potential molecular mechanisms of colchicine-induced autophagic degradation of HBc, HepG2-NTCP cells infected with HBV were subjected to colchicine treatment for RNA sequencing. Twenty-three DEGs were identified (|Log2 FC | >1; *P*-value < 0.05, Fig. 4A), and the DEGs were further subjected to Reactome enrichment analysis. Among these pathways, the activation of AMPK downstream of NMDARs pathway has attracted our attention (Fig. 4B). AMPK plays an important role in autophagy by regulating energy metabolism, and its major function is regulating the autophagy process [19]. Activation of the AMPK pathway can result in the dephosphorylation of mTOR and phosphorylation of ULK1 [19], both of which lead to the activation of autophagic. Therefore, the effect of colchicine on the expression of AMPK and its downstream genes was evaluated. Although the total protein level of AMPK remained stable after colchicine treatment, the phosphorylated AMPK level significantly increased in a dose-dependent manner (Fig. 4C). As expected, our data demonstrated that AMPK activation led to the dephosphorylation of mTOR and phosphorylation of ULK1 (Fig. 4C), indicating that the AMPK/mTOR/ULK1 signalling pathway plays a key role in Col-induced HBc degradation. Then, we transferred GFP-LC3 into the cells and observed an increased number of LC3 puncta in the Col-exposed cells compared with the control cells by immunofluorescence staining (Fig. 4D). Consistent with these findings, Beclin-1 and ATG5 levels and the LC3II/LC3I ratio were increased, which was accompanied by a decrease in p62 levels upon colchicine exposure, and these effects were dose dependent (Fig. 4E), suggesting that colchicine triggered autophagic degradation. In addition, transmission electron microscopy (TEM) revealed that the number of autophagosomes was increased in Col-treated cells (Fig. 4F). Taken together, our data suggest that Col induces autophagy degradation through the AMPK/mTOR/ULK1 signalling pathway.

### Anti-HBV effect of colchicine in vitro

**Fig. 5:**
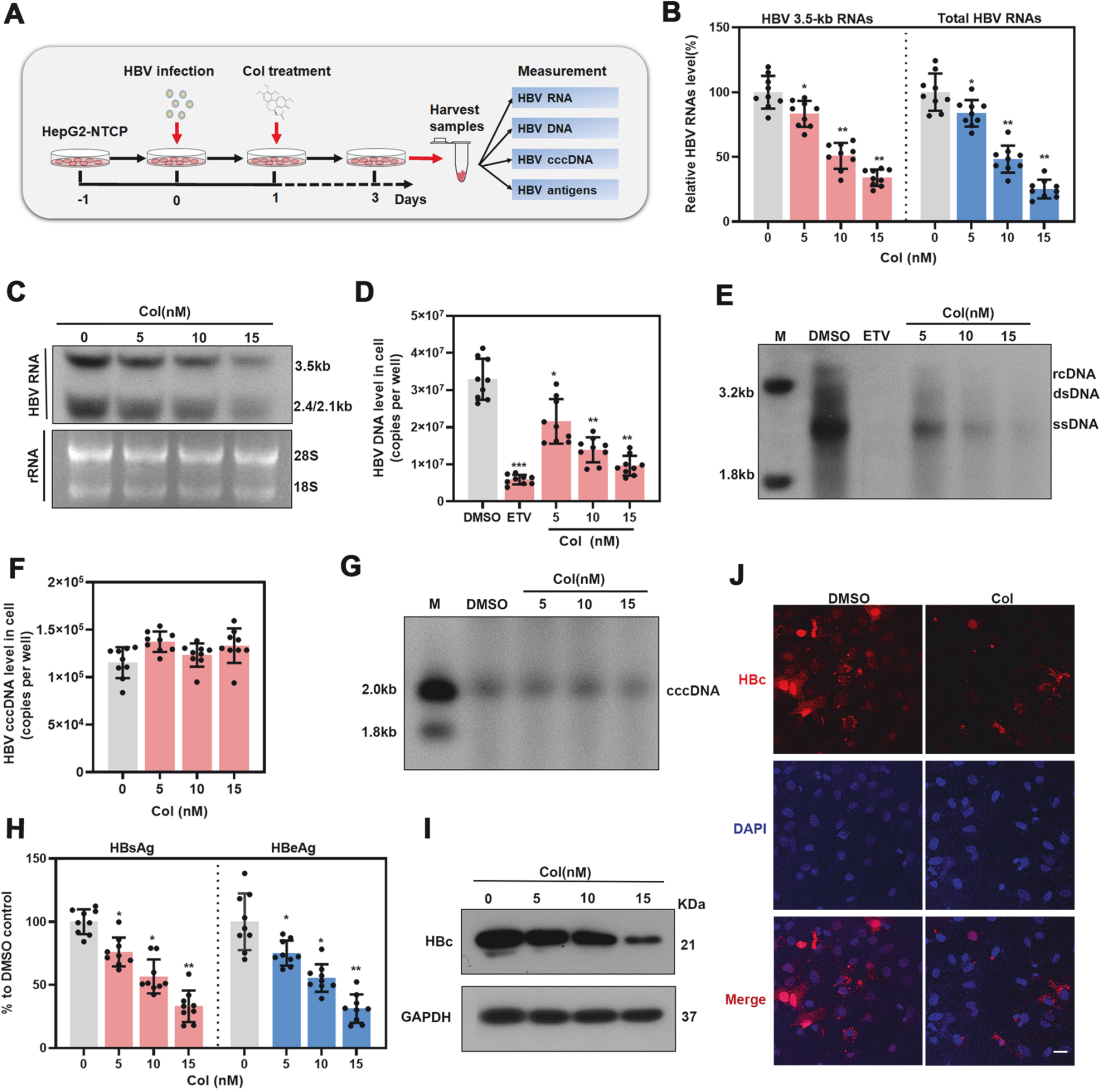
Colchicine inhibited HBV transcription and replication in vitro. **A** Schematic depiction of HBV infection, colchicine treatment and samples collected to detect HBV RNA, DNA, cccDNA and antigens. **B** Real-time PCR detection of HBV RNA levels. **C** HBV RNA was analysed using Northern blot hybridization. **D**, **E** Real-time PCR and Southern blotting for detecting HBV DNA levels. **F**, **G** Taq-Man probe q‒PCR and Southern blotting for detecting HBV cccDNA levels. **H** Collect cell culture supernatant to detect HBsAg and HBeAg levels. **I** Determine HBc protein levels using western blotting. **J** Intracellular HBc was visualized by immunofluorescence staining. Scale bar = 25 *μ*m. The data was expressed as the mean ± SD of three replicates of the experiment. (*P < 0.05; **P < 0.01; * * *P < 0.001).

Since colchicine significantly decreases the level of the HBc protein, which is essential for HBV replication [20], we next evaluated the effect of colchicine on HBV transcription and replication. HepG2-NTCP cells were infected with HBV and treated with different concentrations of colchicine (Fig. 5A). Real-time PCR revealed that colchicine obviously decreased the level of HBV RNA (Fig. 5B), which was further validated by Northern blotting (Fig. 5C). Next, to explore the effect of colchicine on HBV DNA, we used entecavir (ETV), a nucleoside analogue that can effectively inhibit HBV DNA replication [21, 22], as a positive control. Although colchicine was less effective than ETV, HBV DNA levels decreased in a dose-dependent manner after colchicine treatment, as shown by real-time PCR and Southern blotting (Fig. 5D, E). In addition, the level of cccDNA remained stable after colchicine treatment (Fig. 5F, G). Furthermore, we also determined the effect of colchicine treatment on HBV protein levels. The secretion of HBeAg and HBsAg was significantly decreased in colchicine-exposed cells (Fig. 5H). Similarly, the level of HBc was significantly reduced by colchicine treatment, as determined by western blotting and fluorescence staining (Fig. 5I, J). In summary, these results indicated that colchicine has a significant inhibitory effect on HBV.

### Anti-HBV effect of colchicine in vivo

**Fig. 6:**
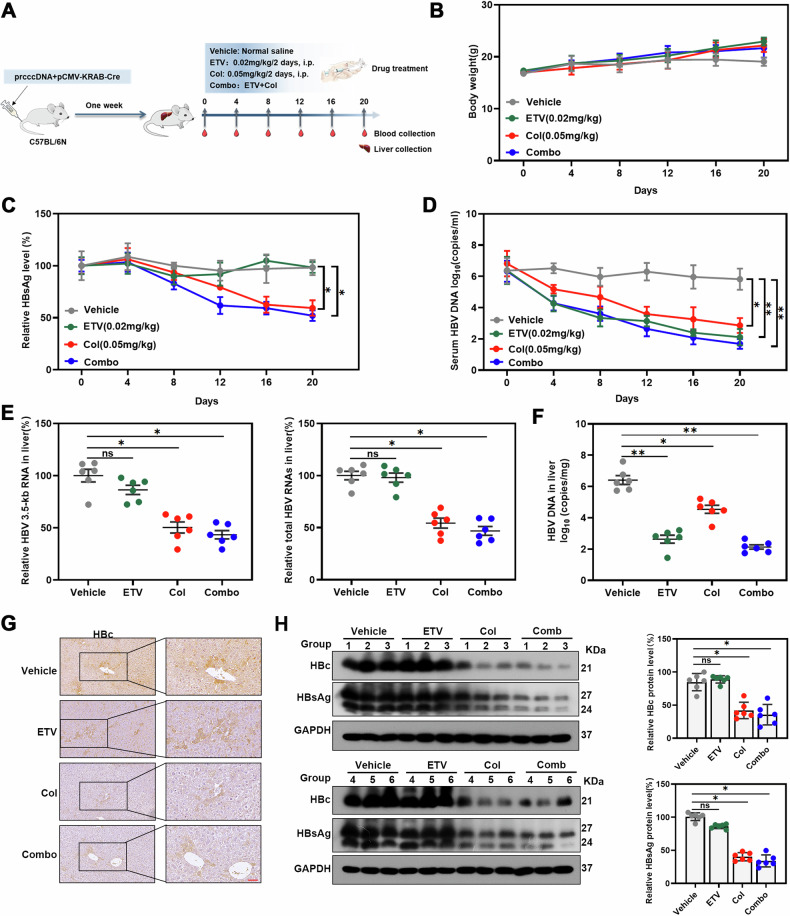
Colchicine inhibited HBV in vivo. **A** Flow chart showing the method and concentration of colchicine and ETV used and the timing of orbital blood collection. **B** Body weight was monitored every 4 days. **C** Detect HBsAg levels in serum using chemiluminescent microparticle immunoassay. **D** Real-time PCR detection of HBV DNA levels in serum. **E** Real-time PCR detection of HBV RNA levels in liver tissue. **F** Real-time PCR detection of HBV DNA levels in liver tissue. **G** Analysis of HBc levels in liver tissue by using immunohistochemistry. Scale bar = 40 μm. **H** Determine HBc and HBsAg protein levels using western blotting. Statistical analyses were performed using Student’s *t* test. (*P < 0.05; **P < 0.01).

Since colchicine plays an effective role in inhibiting HBV in vitro, we further explored whether colchicine could suppress HBV in vivo. First, we assessed the toxicity of Col. Randomly divide the mice into 3 groups (*n* = 6) and then intraperitoneally injected with different concentrations of colchicine (0 mg/kg, 0.025 mg/kg or 0.05 mg/kg) every other day for 20 days. Blood analysis was carried out on day 20. There was no significant difference in toxicity after treatment with colchicine (Table S3). Based on the in vivo toxicity assay results, 0.05 mg/kg colchicine was selected for further study. Moreover, we evaluated the dose by measuring the blood concentration after the administration of 0.05 mg/kg. Since the administration frequency was once every 2 days, pharmacokinetic samples were collected 48 h after the intraperitoneal administration of 0.05 mg/kg colchicine. The Cmax of colchicine was 24.64 ng/mL. In addition, the half‐life (T_1/2_) of colchicine was 0.2 h, which indicates that it has a short half‐life (Fig. S5A). However, considering that the concentration of colchicine for cell use is relatively low and that the detection range of the blood concentration is limited, the plasma concentration of colchicine could not be detected after 4 h. Moreover, considering the trauma to the mice caused by multiple short-term administrations, in subsequent animal studies, we chose to administer the drug on alternate days (Fig. 6A).

To establish an HBV expression mouse model, we used a previously described mouse model of HBV infection. Based on the good effect of entecavir in inhibiting HBV DNA, we combined colchicine and entecavir, and therefore randomly divided mice into 4 groups: the vehicle group, colchicine group, entecavir (ETV) group, and colchicine combined with ETV group (the Combo group). The mice were treated with the indicated drugs every other day, and blood was collected every 4 days. On day 20, the mice were euthanized, and liver tissue was collected for further examination (Fig. 6A). Body weight was monitored, and no differences were found between the groups (Fig. 6B). Based on the results (Fig. 6C–F), we found that the levels of HBsAg and HBV DNA in serum were significantly reduced in the colchicine group (Fig. 6C, D), as were the levels of HBV RNA and HBV DNA in liver tissues (Fig. 6E, F). However, entecavir was more effective than colchicine in inhibiting HBV DNA (Figs. 6D, F). Thus, colchicine combined with entecavir significantly enhanced the inhibitory effect of colchicine on HBV DNA (Fig. 6F). In addition, colchicine treatment resulted in a significant reduction in the intrahepatic HBc level, as evidenced by immunohistochemistry and western blotting (Fig. 6G, H). Similarly, HBsAg levels were significantly decreased, as evidenced by immunohistochemistry and western blotting (Fig. 6H, S5B). In general, we found that colchicine inhibited HBV replication in vivo.

### Colchicine inhibits HBV through the autophagic degradation of HBc

**Fig. 7:**
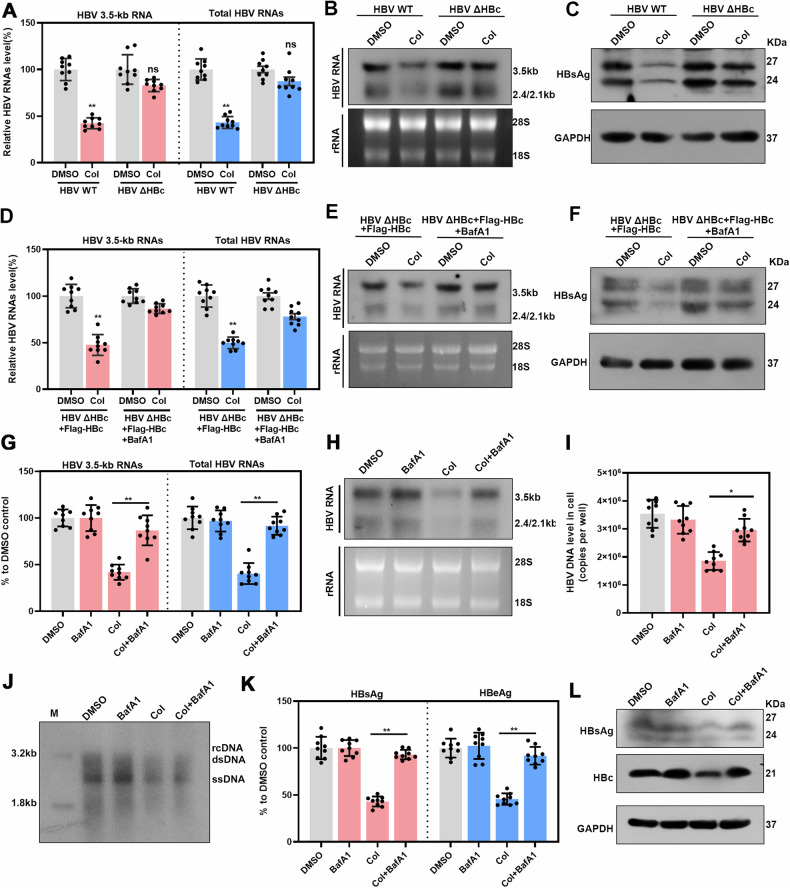
Colchicine inhibits HBV transcription through the autophagic degradation of HBc. **A**–**C** Transfect plasmids encoding HBV WT or HBV ΔHBc into Huh-7 cells and treated with or without Col for 2 days. **A** Real-time PCR detection of HBV RNA. **B** HBV RNA was analysed using Northern blot hybridization. **C** Determine HBsAg protein levels using western blotting. **D**–**F** Transfect plasmids encoding HBV ΔHBc into Huh-7 cells and transfected with plasmid encoding Flag-HBc for 24 h, then treated with or without Col for 24 h, and then treated with or without BafA1 for 24 h. **D** Real-time PCR detection of HBV RNAs. **E** Analyze HBV RNA using Northern blot hybridization. **F** Determine HBsAg protein levels using western blotting. **G**–**L** HepG2-NTCP cells infected with HBV were treated with or without Col for 24 h, and then, the cells were treated with or without BafA1 for 24 h. **G** Real-time PCR detection of HBV RNAs. **H** Analyze HBV RNA using Northern blot hybridization. **I**, **J** Real-time PCR and Southern blotting detection of HBV DNA levels. **K** Collect cell culture supernatant to detect HBsAg and HBeAg levels. **L** HBc and HBsAg levels were determined by western blotting. The data was expressed as the mean ± SD of three replicates of the experiment. (*P < 0.05; **P < 0.01).

To explore whether the impacts of colchicine on HBV transcription and replication are mediated by HBc, we constructed a HBc-deficient plasmid (HBV-ΔHBc) in which the expression of the C gene was abrogated. Huh-7 cells were transfected with the WT HBV or HBV-ΔHBc plasmid, and a series of HBV markers were examined (S6A-B). Our data revealed that colchicine could effectively reduce HBV RNA levels in cells transfected with HBV-WT but failed to reduce the HBV RNA levels in cells transfected with the HBV-ΔHBc plasmid (Fig. 7A, B). Similarly, colchicine did not decrease HBsAg levels under these conditions (Fig. 7C). In addition, Flag-HBc was used to restore HBc levels in cells transfected with the HBV-ΔHBc plasmid, and the effect of BafA1 on Col-mediated HBV inhibition was observed. A clear decrease in the HBV RNA level was observed in cells transfected with Flag-HBc, and this decrease was abolished by BafA1 (Fig. 7D, E). In addition, BafA1 reversed the decrease in HBsAg levels caused by colchicine treatment in cells transfected with Flag-HBc (Fig. 7F). Furthermore, we evaluated whether the inhibition of HBV transcription by colchicine depends on HBc in the HBV infection system. We found that the reduction in HBV RNA levels in Col-treated cells was dramatically attenuated by the autophagy inhibitor BafA1 (Fig. 7G, H). Similarly, the effect of colchicine on HBV DNA was also abolished by BafA1 treatment, as was the effect of HBV DNA on HBeAg, HBsAg and HBc levels (Fig. 7I-L). These data verified that colchicine can inhibit HBV transcription and replication through autophagic degradation of HBc.

### Colchicine inhibits HBc-mediated liver cancer metastasis

**Fig. 8:**
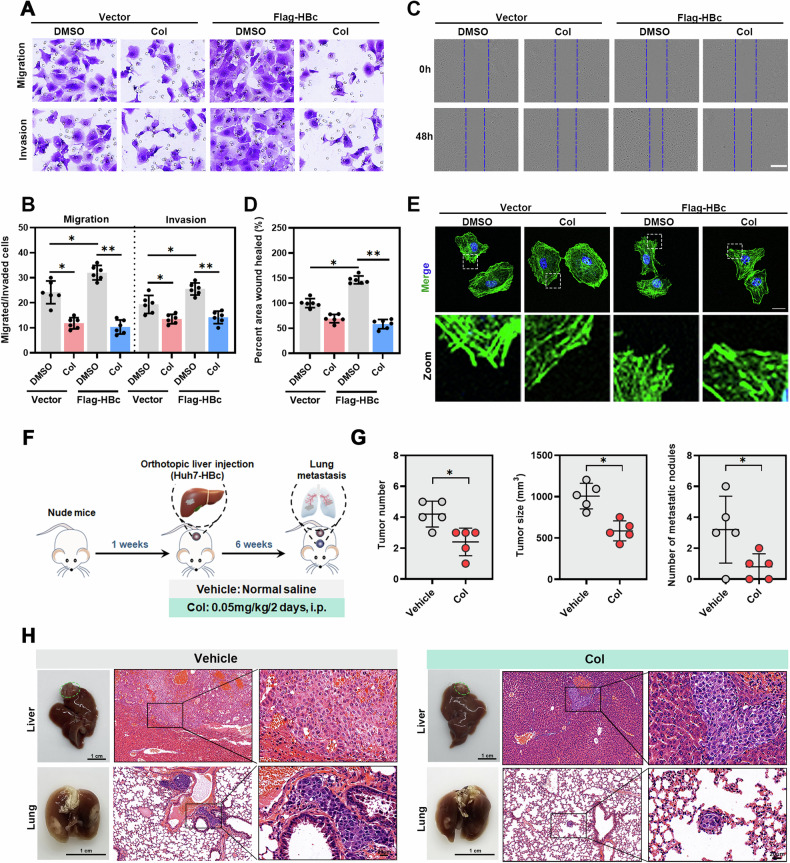
Colchicine inhibits HBc-mediated liver cancer metastasis. **A**–**E** Transfect plasmids encoding Vector (pcDNA3.1) or Flag-HBc into Huh-7 cells and treated with or without Col for 2 days. (A-B) Detect the effect of Col on HCC migration and invasion through transwell. **C**, **D** Detect the effect of Col on the healing velocity through wound healing assay. Measure the distance from 6 images was measured. Scale bar = 400 *μ*m. **E** Phalloidin was used to stain the cytoskeleton, while DAPI was used to label the nuclei of cells. Scale bar = 25 *μ*m. **F** Flow chart showing the method and concentration of colchicine used. The Lung metastasis model was established by orthotopically injecting Huh-7 cells with HBc stably expressed (n = 5). **G** Tumor number, tumor size and metastatic nodules were calculated (*p < 0.05). **H** Representative images of tumor-bearing liver, lung and H&E staining for liver and lung tissues were provided. (*P < 0.05; **P < 0.01).

HBc plays an important role in HCC metastasis and malignant progression [11], and we further investigated the role of colchicine in HCC metastasis. First, we transfected Flag-HBc into Huh-7 cells, and a series of experiments were conducted to determine the effect of colchicine on HBc overexpression on HCC progression. We found that colchicine significantly decreased the migration, invasion and wound healing capacity of Huh-7 cells overexpressing HBc compared with Huh-7 cells transfected with Vector (Fig. 8A–D). In addition, F-actin staining revealed that colchicine treatment significantly reduced the formation of lamellipodia in Huh-7 cells overexpressing HBc (Fig. 8E).

To investigate the effect of colchicine on HCC metastasis in vivo, we constructed Huh7 cells that stably expressed HBc (Fig. S7A). Huh7-HBc or Huh7-Vector cells were injected into the left lobe of the liver of nude mice to generate an in situ nude mouse HCC model. The mice were treated with the indicated drugs every other day. After 6 weeks, the mice were euthanized, and liver and lung tissues were collected for analysis (Fig. 8F and S7B). We found that colchicine was effective in reducing the number and size of liver tumor in mice injected with Huh7-HBc cells compared with those injected with Huh7-Vector (Fig. 8G and S7C). In addition, in mice injected with Huh7-HBc cells, the number of lung metastases in the colchicine-treated mice was significantly lower than that in the control mice (Fig. 8G, H), which was not observed in mice injected with Huh7-Vector (Fig. S7C, D). Taken together, these data confirm that colchicine can inhibit HBc-associated metastasis both in vivo and in vitro.

## Discussion

HBc is one of the key viral proteins of HBV, and HBc plays an important role in the HBV lifecycle, including viral capsid formation, reverse transcription and viral mini-chromosome formation [8]. Although some studies have shown that the absence of HBc may have little effect on HBV RNA and protein [23], we found that the level of HBV RNA decreased at 5-7 days after HBV-ΔHBc transfection (Fig. S6A), suggesting that the absence of HBc may hindered viral packaging and replication, formation of new viral particles, and completion of the viral life cycle. Because of the important role of HBc, targeting HBc has become a research hotspot in recent years. After the discovery of BAY41-4109, a heteroaryldihydropyrimidine (HAP) derivative [24], and AT130, a phenylpropenamide derivative [25], Compound targeting capsid assembly (CAMs) appear promising. However, because of safety and pharmacological issues, CAM treatment has not yet entered the clinic. In addition, increasing evidence indicates that the HBc protein can induce malignant proliferation and metastasis of cancer cells. A study has elucidated HBc facilitates HCC cell migration, invasion, and cytoskeleton regulation via the miR-382-5p/DLC-1 axis [26]. Furthermore, HBc also plays a multifaceted role in the occurrence and progression of liver cancer. In addition to its impact on signaling pathways related to cancer cell migration and proliferation, it also impacts apoptotic pathways and cellular metabolic pathways during HCC development. HBc can interact with the immune system, shaping the tumor microenvironment through the expression and production of proinflammatory cytokines [11]. Therefore, the development of compounds that can inhibit HBc is urgently needed.

Colchicine is a natural alkaloid compound derived from *Colchicum autumnale* [27] that was initially used as an effective compound for the treatment of gout and familial Mediterranean fever (FMF), and its pharmacokinetics have been well studied [28]. After oral administration of 0.6 to 1 mg of colchicine in humans, its peak plasma concentrations range from approximately 2 to 6 ng/ml [29, 30]. Moreover, colchicine is selectively distributed in the liver and kidney [31] and has been reported to have anticancer effects on HCC at low concentrations [32]. In addition, micromolar concentrations of colchicine and its derivatives have been shown to inhibit HIV [33], dengue virus and Zika virus in cell culture [34]. The major pharmacological disadvantage of colchicine in therapy is its high cytotoxicity. Therefore, in cell experiments, we found that colchicine could exert potent anti-HBV and HBV-related effects on HCC at lower doses (15 nM). On the other hand, in the mouse experiment, we tightly controlled the concentration of colchicine (0.05 mg/kg) based on the pharmacokinetic results. Therefore, the clinically acceptable concentration of colchicine may inhibit HBV infection and HBc-HCC metastasis, suggesting that colchicine is a potential anti-HBV and HBV-related HCC drug.

AMPK/mTOR/ULK1 pathway plays an important role in the regulation of autophagy [35]. Some studies have reported that activated AMPK can increase the levels of Beclin-1 [36] and ATG5 [37], further promoting the autophagic process. Our study also found that autophagy markers were significantly upregulated after activation of AMPK/mTOR/ULK1 signaling pathway by colchicine treatment. Autophagy plays a crucial role in regulating natural antiviral immune responses and host immunity [38, 39]. Several studies have reported the important role of autophagy in HBV infection, whether selective autophagy plays a key role in HBV infection remains unclear. Selective autophagy is a form of autophagy in which labelled substrates are recognized by cargo receptors for degradation signals and then delivered to the autophagic membrane through the LC3 interaction region (LIR) [40]. Therefore, is the molecular mechanism by which colchicine inhibits HBc related to the autophagic degradation of HBc? Our results suggest that colchicine promotes the binding of HBc to the autophagy receptor p62, leading to the recruitment of p62 and LC3 to initiate autophagy. Subsequently, ubiquitination-dependent autophagic degradation occurs. However, GST pull-down confirmed that p62 could not bind to HBc directly, as protein ubiquitination is a complex process involving Ubiquitin-activating enzyme (E1), ubiquitin-conjugating enzyme (E2), and ubiquitin ligase (E3), of which E3 is essential for target protein recognition [41, 42]. Therefore, further studies are needed to prove whether p62 binds to E3 and regulates ubiquitination and degradation of HBc. Taken together, our findings provide evidence that colchicine inhibits HBc by inducing selective autophagic degradation of HBc.

Based on the inhibitory effect of colchicine on HBc, colchicine plays a role in the disease progression of both HBV and HBV-related HCC. However, several issues were not well elucidated in our research, and colchicine appeared to have no significant effect on cccDNA levels. Colchicine inhibits cell division by promoting microtubule depolymerization [43], and cell division appears to cause cccDNA dilution among larger cells and intrahepatic cccDNA loss [44]. Therefore, colchicine inhibits the loss of cccDNA to some extent, which contradicts our findings that colchicine inhibits cccDNA transcription. However, further research is needed to determine the role of colchicine in the stability of cccDNA. In addition, for the clinical application of colchicine, efforts to achieve a longer half-life and lower toxicity are needed. Future studies may consider drug modification to extend the half-life of colchicine or the use of new drug delivery methods to improve its targeting and toxicity and reduce its dosage. Finally, in the HCC metastasis study, we found that colchicine was more effective in inhibiting the HBc-associated metastasis. However, both cellular and animal experiments have found that colchicine itself may have an impact on the migration and proliferation of HCC (Fig. 8A–E and S7B––D). Therefore, the mechanisms need further research. For example, colchicine has been shown to inhibit HCC proliferation and tumor growth [45]; colchicine promotes the anticancer effects of sorafenib or regorafenib on hepatocellular carcinoma [32]. Does colchicine inhibit liver cancer progression through other mechanisms? This requires further exploration.

Overall, our study establishes a dual luciferase reporter assay and identifies that colchicine inhibits HBc expression, and further mechanistic studies showed that colchicine promotes the selective autophagic degradation of HBc. Our study suggested that colchicine is a potential drug candidate for the treatment of HBV and HBV-related HCC by targeting the HBc protein.

## Materials and methods

### Cell culture

The HepG2 liver cancer cell line was purchased from ATCC (HB-8065), and the Huh-7 liver cancer cell line was obtained from the Japanese Collection of Research Bioresources (JCRB0403). HepG2-NTCP liver cancer cells were derived from HepG2 cells and constructed in our laboratory according to a previous study [46]. The three cell lines were cultured in DMEM (D6429, Sigma‒Aldrich, Germany) supplemented with 10% fetal bovine serum (FBS) (10270, Corning, New York, USA) and 1% penicillin/streptomycin (PS) (Thermo). HepAD38 cells, which had integrated HBV DNA composed of a 1.1-fold excess of the HBV genome, were kindly provided by Professor Ning-Shao Xia (Xiamen University, China) and maintained in DMEM (D6429, Sigma‒Aldrich, Germany) containing 10% FBS, 1% PS and 400 μg/mL G418 (Merck). All the cell lines were verified by short hairpin RNA (STR) DNA testing, regularly tested for mycoplasma contamination and confirmed to be mycoplasma free. The cell STR profiles are available upon request from the authors.

### Plasmid and transfection

The plasmid encoding GFP-LC3 was donated by Professor Yong Lin. The plasmid encoding GFP-p62 was purchased from YouBio (China, #34662). pCH9/3091 was donated by Professor Lin Lan (The Army Medical University, Chongqing, China). The full-length sequences of HBc, HBx, HBs and HBp were inserted into pcDNA3.1 to construct plasmids expressing different HBV viral proteins, and the C-terminus of pcDNA3.1 contained a Flag tag. pCH9/3091-ΔHBc, containing a stop codon mutation in the HBc gene (codon 40), was obtained from Professor Jeli Hu (Chongqing Medical University, China). The pGL3-HBc plasmid was constructed by cloning HBc into the firefly luciferase pGL3 reporter plasmid (Promega, USA). The plasmids were transfected using Lipofectamine™ 3000 (Invitrogen, United States). Briefly, plasmid DNA-lipid complexes were prepared from 1.25–5 μg/μL of DNA with P3000™, Opti-MEM™ Medium and Lipofectamine™ 3000 reagent. After incubation for 10–15 min at room temperature, DNA-lipid complexes were added to the cells.

### Cell cytotoxicity assay

Cell viability was measured using MTT (3-(4,5-dimethylthiazol-2-yl)−2,5-diphenyltetrazolium bromide) (Sangon Biotech). Briefly, cells were treated with different concentrations of compounds, 5 mg/mL MTT was added to each well for 4 h, 100 μL of DMSO was added to each well, and the absorbance at 490 nm was measured by a microplate reader. The 50% cytotoxicity concentrations (CC_50_) were calculated via nonlinear regression using GraphPad Prism.

### Dual-luciferase reporter assay

The cells were transfected with luciferase reporter plasmids and treated with the compound for 48 h, and Renilla plasmids were cotransfected as an internal control. Finally, luciferase activity was measured using a GloMax microplate spectrophotometer (Promega, USA).

### RNA interference

Small interfering RNAs (siRNAs) targeting p62 (NS-005579-001) and ATG5 (NS-006885-001) and a negative control siRNA (NS-010424-001) were designed and synthesized by Tsingke (Chongqing, China). For siRNA transfection, the cells were seeded and transfected with each siRNA (0.25 μM) at 37 °C for 1 day using Lipofectamine™ 3000. The siRNA sequences are shown in Table S1.

### Virus preparation and infection

HBV particles were collected from the culture supernatant of HepAD38 cells. Briefly, the cell culture supernatant was collected and mixed with polyethylene glycol (PEG) 8000. Then, the HBV particles were collected by gentle rotation overnight at 4 °C and centrifugation at 4000 rpm for 30 min at 4 °C. The centrifuged precipitate was dissolved in Opti-MEM.

For HBV infection, HepG2-NTCP cells were infected with HBV (1 × 10^3^ genome equivalents/cell) for 24 h in the presence of 4% PEG8000. The next day, the cells were washed and maintained in culture medium supplemented with 10% FBS and 2.5% DMSO.

### ELISA

An ELISA kit (KHB, China) was used to measure the levels of HBsAg and HBeAg in the culture supernatant.

### Determination of serum HBsAg

An Abbott Architect System with HBsAg Reagent Kit (08P0877, Abbott, Chicago, USA) was used to detect HBsAg levels in the serum.

### Modified hirt cccDNA extraction

Cell samples were lysed in lysis buffer (50 mM Tris HCl, 10 mM EDTA, 150 mM sodium chloride, 1% SDS) for 20 min. Then, KCl was added to the cell lysate, and the mixture was incubated overnight at 4 °C. The next day, the supernatant was collected by centrifuging the lysate twice at 12000 × *g* for 20 min, and the DNA was extracted with phenol/chloroform (1:1) before adding isopropanol to precipitate the DNA. Then, the DNA precipitate was washed with 70% ethanol, and Taq Man probe q-PCR and Southern blotting were used to detect the expression of HBV cccDNA in the samples. All primers and probes used are listed in Supplementary Table S1.

### Purification of HBV DNA

Cell samples and liver tissues (10–20 mg) were lysed in lysis buffer at 37 °C for 15 min as previously described [47]. After centrifugation of the sample, the supernatant was collected, 10 mM MgCl_2_ and 40 IU/mL DNase I were added, and the mixture was incubated at 37 °C for 4 h. After digestion was stopped by the addition of 25 mM EDTA, 0.5 mg/mL proteinase K and 1% SDS were added, and the mixture was incubated overnight at 45 °C. The next day, the supernatant was extracted with phenol/chloroform (1:1), and isopropyl alcohol was added to precipitate the DNA. Then, the DNA precipitate was washed with 70% ethanol. Finally, absolute quantitative PCR was performed on the HBV DNA levels using Fast Start Universal SYBR Green Master MixT (06924204001, Roche, Mannheim, Germany). Serum HBV DNA was extracted with a TIANamp Virus DNA/RNA Kit (DP315, TIANGEN) and quantified as described above. All primers and probes can be found in Supplementary Table S1.

### Extraction and quantification of HBV RNA

Total RNA was extracted from cells and liver samples (10 ~ 20 mg) with TRNzol reagent (TIANGEN, Beijing, China), after which the RNA was reverse transcribed. After quantification with Fast Start Universal SYBR Green Master Mix, the β-actin mRNA transcript levels were used as an internal control. The expression levels were analysed by the 2-^ΔΔCt^ method, and all primers and probes used are listed in Supplementary Table S1.

### Southern blotting and Northern blotting

DNA, cccDNA and RNA samples were separated on an agarose gel and transferred to a nylon membrane overnight (Roche, Germany). Then, the sample was fixed onto the membrane using a UV crosslinking agent. A DIG high primer DNA labelling and detection starter kit (11585614910, Roche, Mannheim, Germany) was used for DNA hybridization and detection. A DIG Northern Starter Kit (12039672910, Roche, Mannheim, Germany) was used to detect total RNA. 18S and 28S ribosomal RNA were used as loading controls.

### RNA-seq analysis

TRNzol reagent (TIANGEN, Beijing, China) was used for total RNA isolation. According to TruSeq ™ RNA Sample Preparation Guidelines, Using TruSeq ™ RNA sample preparation kit (Illumina, USA) for synthesizing paired-end libraries. Using Qubit®2.0 fluorometer (Life Technologies, USA) to quantify the purified libraries, and the results were validated using an Agilent 2100 Bioanalyzer (USA) to determine the insertion size and calculate the molar concentration. Libraries were diluted to 10 pM using cBot for clustering and then sequenced on an Illumina NovaSeq 6000 (USA) using a PE150 strategy. Library construction and sequencing were performed by Sinotech Genomics Co., Ltd. (Shanghai, China).

### Western blotting

Cell samples and liver tissues (10–20 mg) were lysed, and equal amounts of protein were denatured after detection of the protein concentration using a BCA protein assay kit (Thermo Scientific). Separate protein samples by SDS-PAGE and transfer them on polyvinylidene fluoride (PVDF) membranes (GE Healthcare, Buckinghamshire, UK). After incubation with the corresponding primary antibody at 4° overnight and secondary antibody at room temperature for 2 h. Visualization was performed using an enhanced chemiluminescence (ECL) detection kit (Millipore, Massachusetts, USA). GAPDH was used as a loading control, and the band intensity was calculated by ImageJ software. The antibodies used in this study are listed in Table S2. The original western blots were shown in Supplementary Material 2.

### Co-immunoprecipitation (Co-IP) and GST pull-down

To prevent HBc degradation, cells were treated with 0.1 µM bafilomycin A1 for 24 h before harvest. Then, an appropriate amount of Co-IP lysis buffer (containing protease inhibitors) was added after the cells were washed with precooled PBS, and the cells were lysed on ice for 20 min. Then, total protein, antibody, and anti-FLAG M2 affinity gels (Sigma‒Aldrich, Germany) were gently rotated together overnight at 4 °C. The next day, the supernatant was discarded. The unbound proteins in the beads were washed with protein lysate and then resuspended in 40 µL of 1× loading buffer. The protein complexes were analysed by western blotting.

GST pull-down analysis was performed using the GST pull-down kit (IK-2004, Biolinkedin, Shanghai, China). In brief, His-tag HBc protein (HY-P74354, MedChemExpress, NJ, USA), and the GST-tag p62 protein (Ag13131, proteintech, China) or the GST-controlled protein (HY-P70270, MedChemExpress, NJ, USA) were mixed in a binding buffer containing glutathione-agarose beads and slowly rotation at 4° overnight. Subsequently, the unbound proteins in the beads were washed with protein lysate and then resuspended in 50 µL of 1× loading buffer. The protein complexes were analysed by western blotting.

### Immunofluorescence staining

Fix the cell sample in 4% paraformaldehyde and permeabilize the cells with 0.5% Triton X-100. After blocking for 1 h, incubate the cells with primary antibodies overnight at 4 °C. The next day, the cells were incubated with Alexa Fluor 594 (1:1000) secondary antibodies at room temperature for 2 h. Then, the cells were stained with 4’,6-diamino-2-phenylindole (DAPI) at room temperature and sealed with FluorSaveTM reagent (Millipore, USA, 345789). Images were captured using a laser scanning confocal microscope (LEICADMi8, Germany).

### Immunohistochemistry

Liver tissue from the model mice was used to prepare paraffin-embedded sections. After deparaffinization, tissue antigens were restored with sodium citrate buffer (10 mmol/L, pH 6.0). Then, permeabilization was performed using 0.5% Triton X-100 (Sigma), followed by blocking with 5% BSA for 1 h. DAB staining was performed after incubation with primary and secondary antibodies under the indicated conditions. The nuclei were further counterstained with hematoxylin.

### Transmission electron microscopy

The cells were prefixed with 3% glutaraldehyde, dehydrated with acetone at different concentrations, infiltrated with Epox 812, and embedded. Ultrathin sections were cut with a diamond knife and stained with uranyl acetate and lead citrate. Finally, the sections were viewed with a JEM-1400-FLASH transmission electron microscope.

### Mouse model

Male C57BL/6 N mice (4-5 weeks old; *n* = 66; weight, 16.5–17.9 g) were purchased from the Laboratory Animal Center of Chongqing Medical University. The mice were housed under standard laboratory conditions (light cycle, 12 h of darkness/12 hours of light exposure; temperature, 20 ± 2 °C; humidity, 55 ± 5%), and food and water were freely available.

To assess the toxicity of colchicine. randomly divide the mice into 3 groups (*n* = 6) and then intraperitoneally injected with different concentrations of colchicine (0 mg/kg, 0.025 mg/kg and 0.05 mg/kg) every other day for 20 days. Diet consumption, water intake, body weight, behaviour and health status were monitored every 2 days for all the experimental animals. After 20 days of the experiment, the mice were anaesthetized by intraperitoneal injection of 30 mg/kg pentobarbital sodium. Blood samples (0.2 mL) were collected from the posterior orbital venous plexus for blood analysis using an IDEXX ProCyte Dx analyser (IDEXX Laboratories, Inc., ME, USA).

To assess the pharmacokinetic parameters of colchicine in plasma, randomly divide the mice into eight groups (*n* = 3). After intraperitoneal injection of colchicine (0.05 mg/kg), blood samples were collected at 0.25 h to 48 h, with 3 mice at each time point. Then, the mice were anaesthetized by intraperitoneal injection of 30 mg/kg pentobarbital sodium until deep anaesthesia was achieved. Blood samples (50 µL) were collected from the posterior orbital venous plexus. A 20 µL plasma sample was precipitated with 400 µL of MEOH containing 100 ng/mL IS. After centrifugation, the supernatant was collected and analysed by LC‒MS/MS (TQ6500).

To establish an HBV expression mouse model, 4 µg of precursor recombinant cccDNA (prcccDNA) and the pCMV-KRAB-Cre plasmid (containing a KRAB-Cre fusion recombinase) were injected hydrodynamically into mice through the tail vein for 6–8 s [48]. One week later, randomly divide mice into four groups (*n* = 6): the negative control group (Vehicle), positive control group (ETV, 0.02 mg/kg), colchicine treatment group (Col, 0.05 mg/kg) and Combo group (ETV, 0.02 mg/kg and Col, 0.05 mg/kg). The drugs were administered via intraperitoneal injection. Diet consumption, water intake, body weight, behaviour and health status were monitored every 2 days for all the experimental animals. The frequency of blood collection is shown in Fig. 6A. Mice were anaesthetized by intraperitoneal injection of 30 mg/kg pentobarbital sodium until deep anaesthesia was achieved. Blood samples (70 µL) were collected from the posterior orbital venous plexus. After 20 days of the experiment, 6 mice from each group were euthanized for assessment of the liver.

### In vitro assays for migration and invasion

After culturing the cells with 5 μg/mL mitomycin C for 2 h, the serum-free cultured cells were seeded in the Transwell upper chamber (BD Biosciences), and medium containing FBS was added to the lower chamber. The cells that migrated or invaded were stained with 0.1% crystal violet. Six random fields (40×) were counted under an inverted microscope.

### Wound healing assay

The cells were seeded into well plates and grown to 80% confluence. After 2 h of treatment with 5 µg/mL mitomycin-C, homogeneous wounds were formed with a pipette. After washing three times with PBS, 2 mL of growth medium was added. Images of the scratches were captured by an inverted microscope. The migration rate was calculated by determining the ratio of the wound width at 48 h to the original wound width at 0 h.

### F-actin staining

The cells were seeded onto slides and cultured for 24 h. The cells were fixed with 4% formaldehyde, permeated with 0.5% Triton X-100, and finally stained with DyLight™488 phanxin (CST, #12935) for 15 min and DAPI for 10 min. A laser scanning confocal microscope (Leica DMi8, Germany) was used to capture the images.

### Lentivirus production and cell transduction

The HBc sequence was cloned into the lentiviral vector pCDH-EF1-copGFP-T2A-Puro (Addgene, #72263), and the empty vector was used as a negative control. For transfection, the DNA solution was made up by mixing plasmids of lentiviral vector plasmids with HBc, pMD2. G and psPAX2. Then, HEK-293T cells were cotransfected using Lipofectamine 3000. After collecting the supernatant containing the virus and calculating the virus titre, the cells were infected with 2 mL of the viral supernatant, followed by puromycin (2 μg/mL) selection for 2 weeks.

### In vivo orthotopic mouse model of HCC

Male BALB/C nude mice (4-5 weeks old) were purchased from Byrness Weil Biotech Ltd. A total of 2 × 10^6^ Huh7-HBc and Huh-Vector were resuspended in a mixture of serum-free DMEM/Matrigel (BD Biosciences) and implanted in situ into the left liver lobe of nude mice. After 6 weeks, the mice were anaesthetized by intraperitoneal injection of 30 mg/kg pentobarbital sodium. Primary tumor volume and lung metastasis data were collected for scoring.

### Statistical analysis

GraphPad Prism software was used for statistical analysis. Student’s *t-*test was used to compare differences between two groups. One-way ANOVA was used to compare three or more groups. All the data are expressed as the mean ± standard deviation (SD). Differences of *P* < 0.05 were considered statistically significant (*P < 0. 05, **P < 0. 01, ***P < 0. 001).

### Supplementary information

Figure S1

Figure S2

Figure S3

Figure S4

Figure S5

Figure S6

Figure S7

Supplemental Material

## Data Availability

The dataset used during the current research period has been stored in the NCBI Sequence Reading Archive (SRA) database (login number: PRJNA1012005).
