# Supplementary figures and images for: Colchicine-mediated selective autophagic degradation of HBV core proteins inhibits HBV replication and HBV-related hepatocellular carcinoma progression
Source: Cell Death Discov. 2024 Aug 6;10:352. doi: 10.1038/s41420-024-02122-z (PMC11303544; doi:10.1038/s41420-024-02122-z)

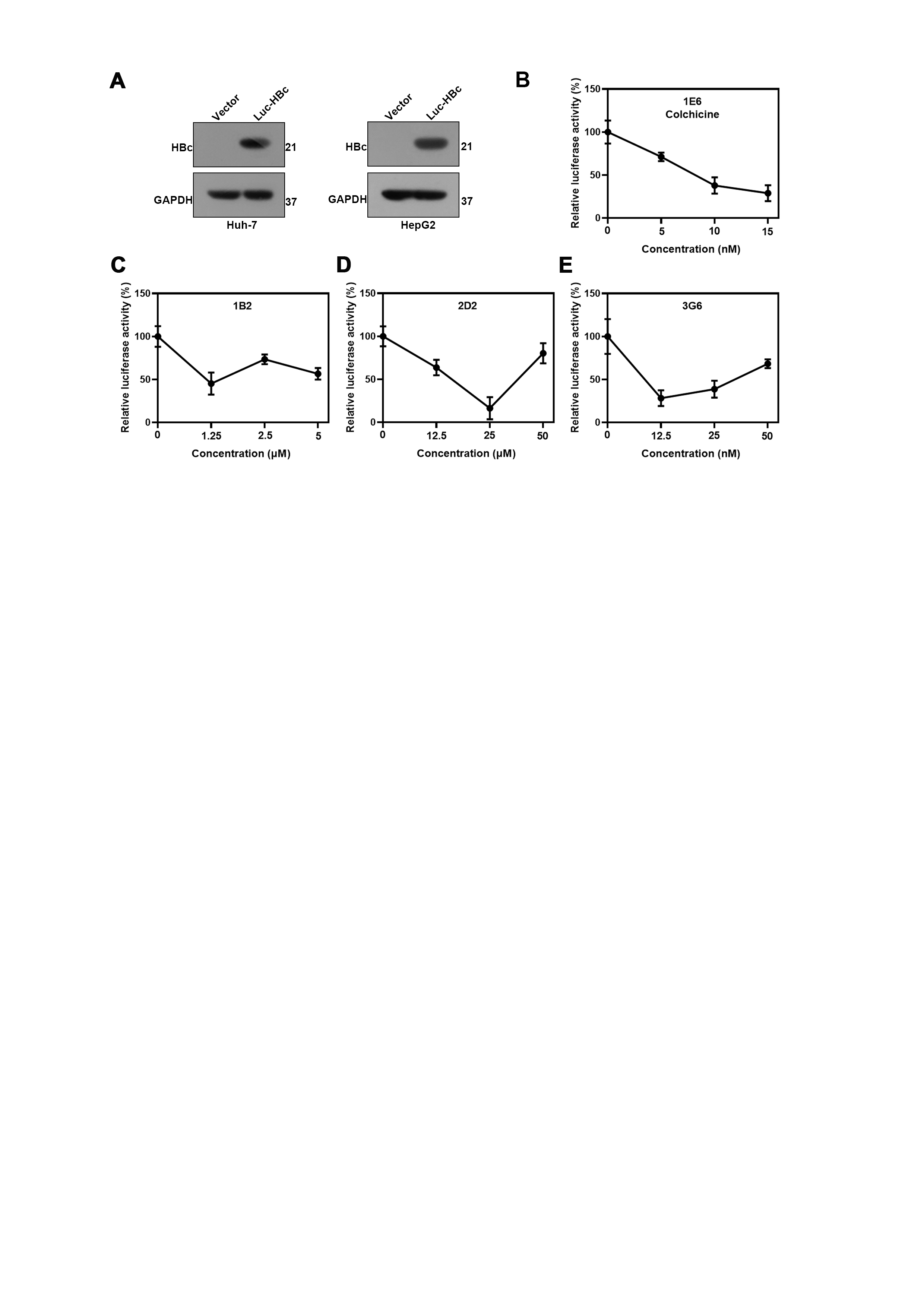

Supplement: Supplementary file 1 — Figure S1 [file 41420_2024_2122_MOESM1_ESM.tif]

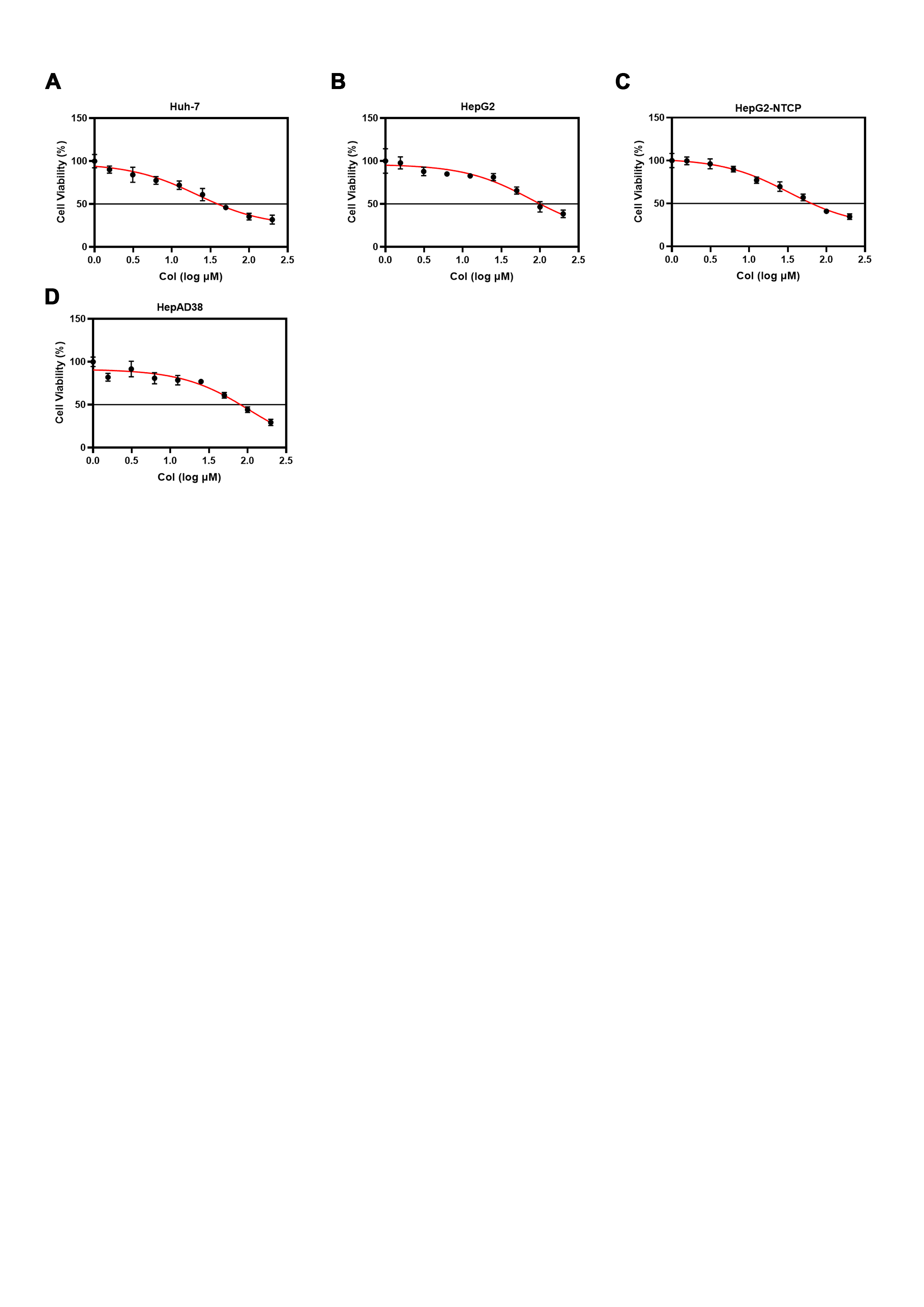

Supplement: Supplementary file 2 — Figure S2 [file 41420_2024_2122_MOESM2_ESM.tif]

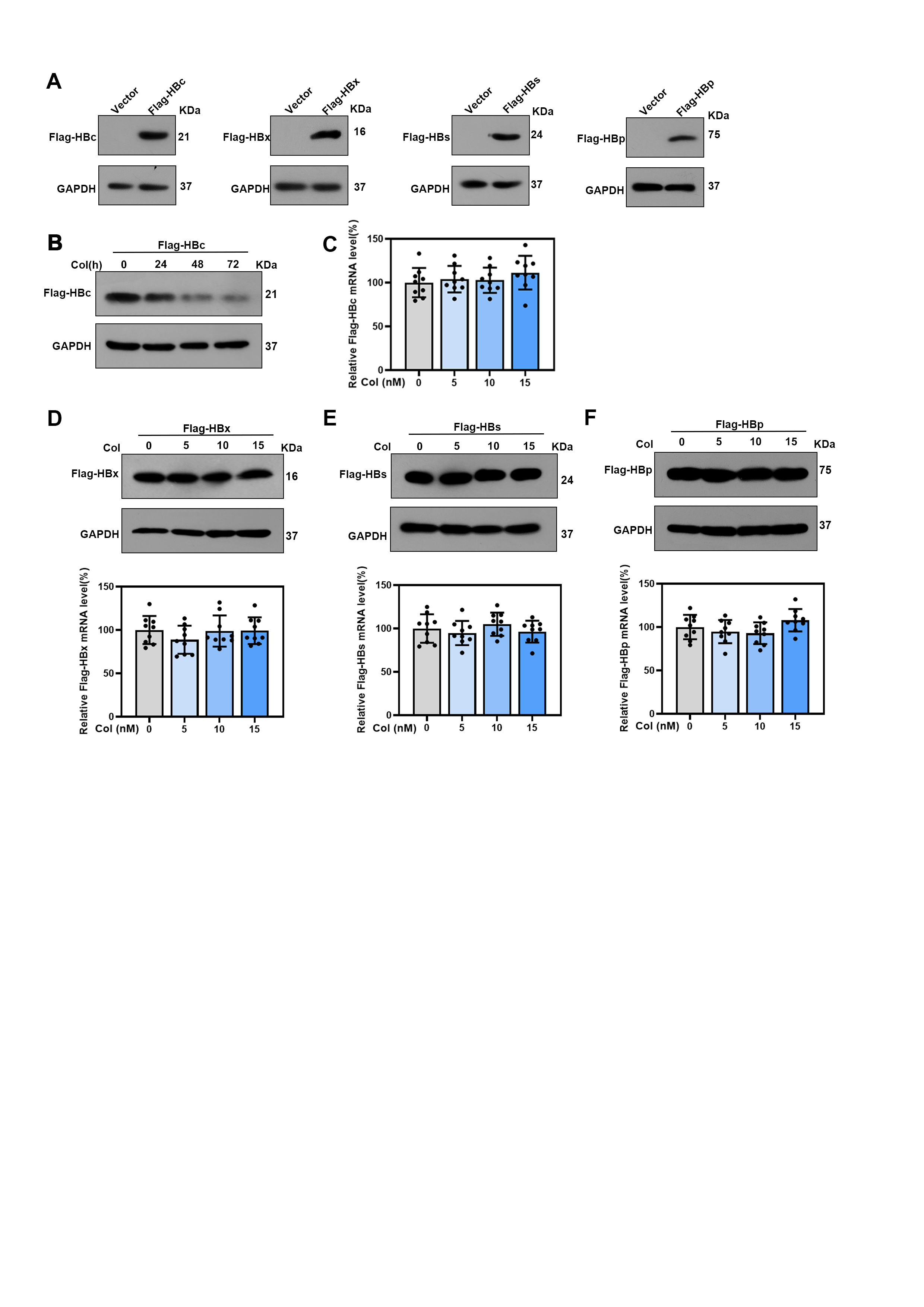

Supplement: Supplementary file 3 — Figure S3 [file 41420_2024_2122_MOESM3_ESM.tif]

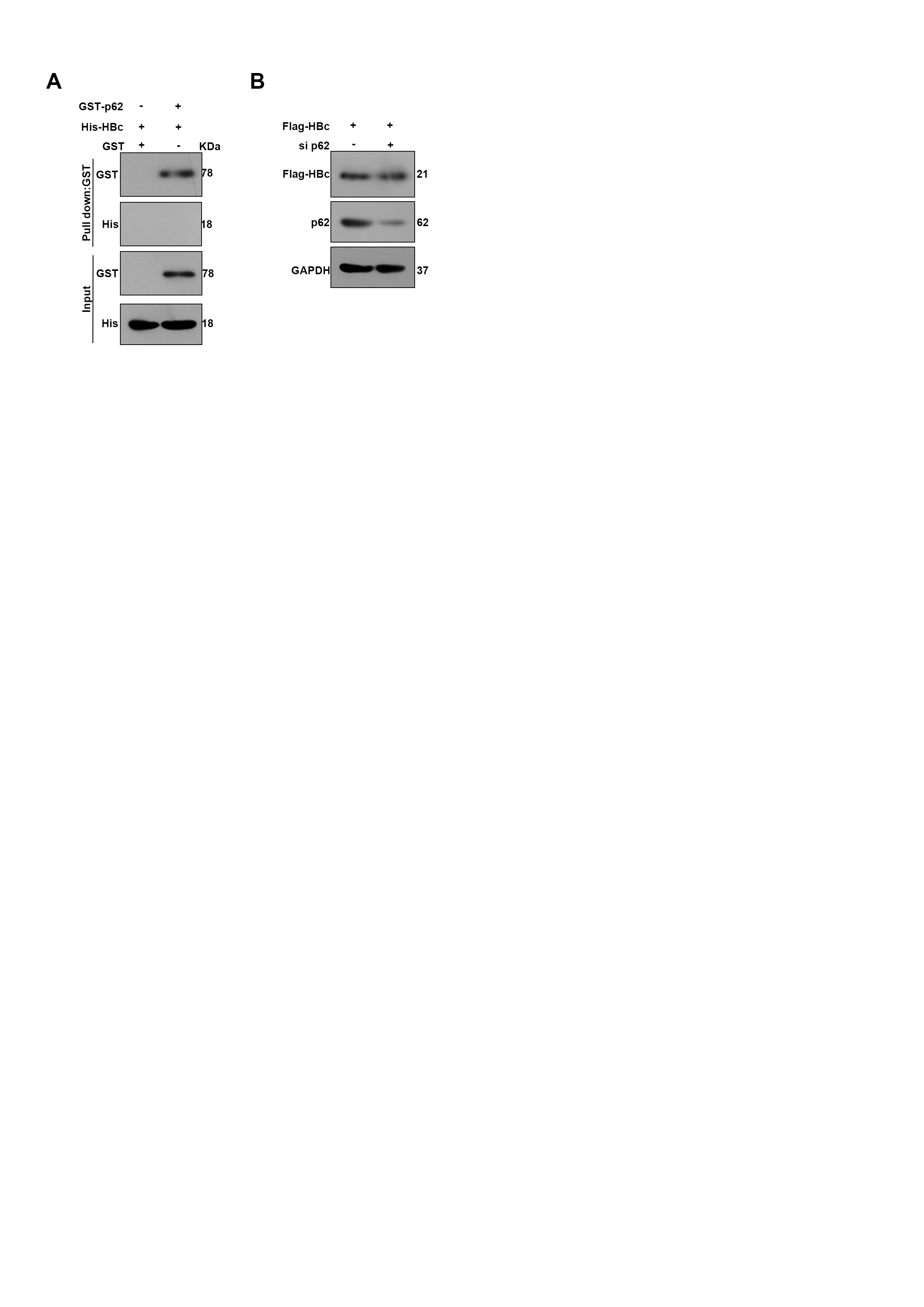

Supplement: Supplementary file 4 — Figure S4 [file 41420_2024_2122_MOESM4_ESM.tif]

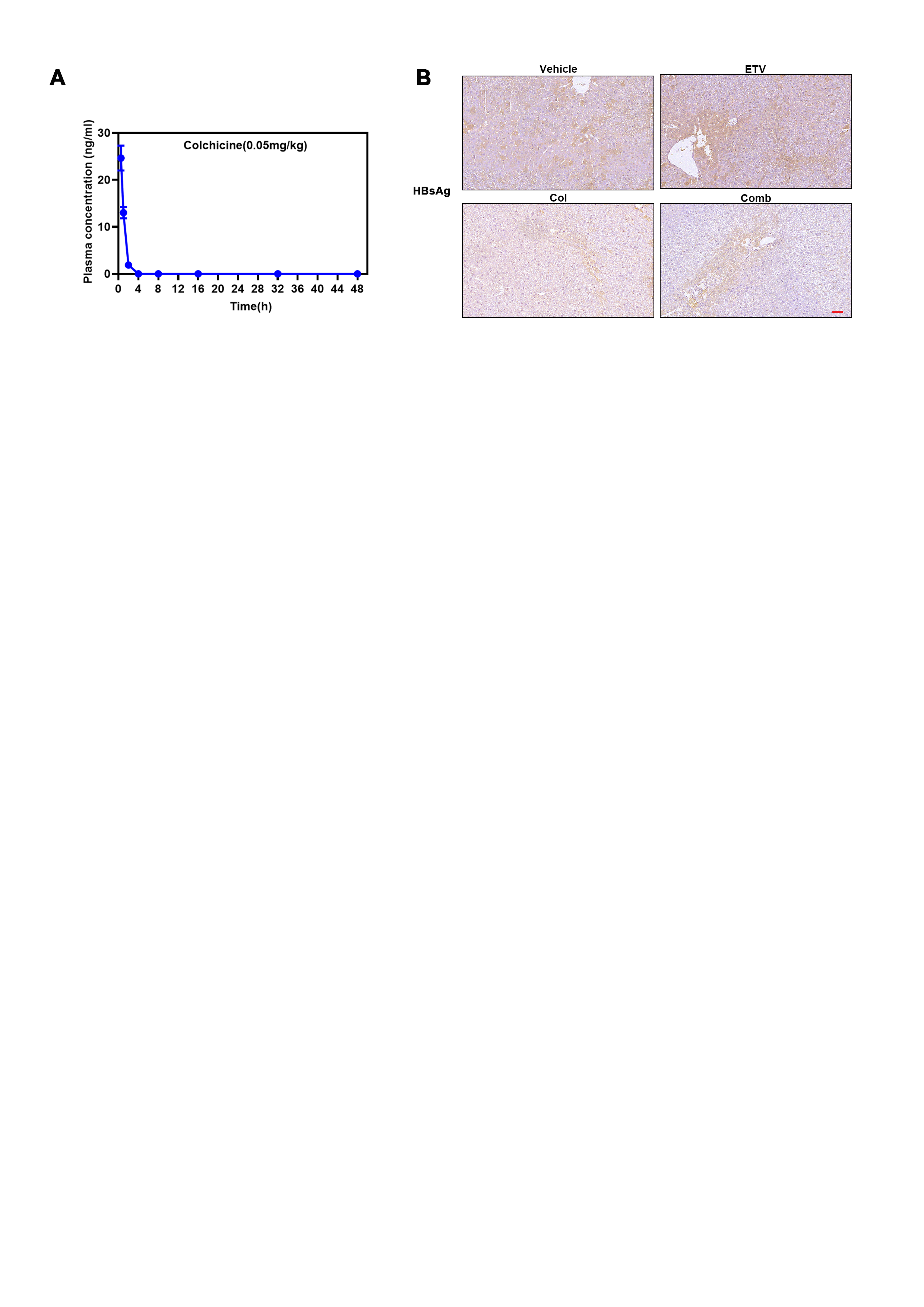

Supplement: Supplementary file 5 — Figure S5 [file 41420_2024_2122_MOESM5_ESM.tif]

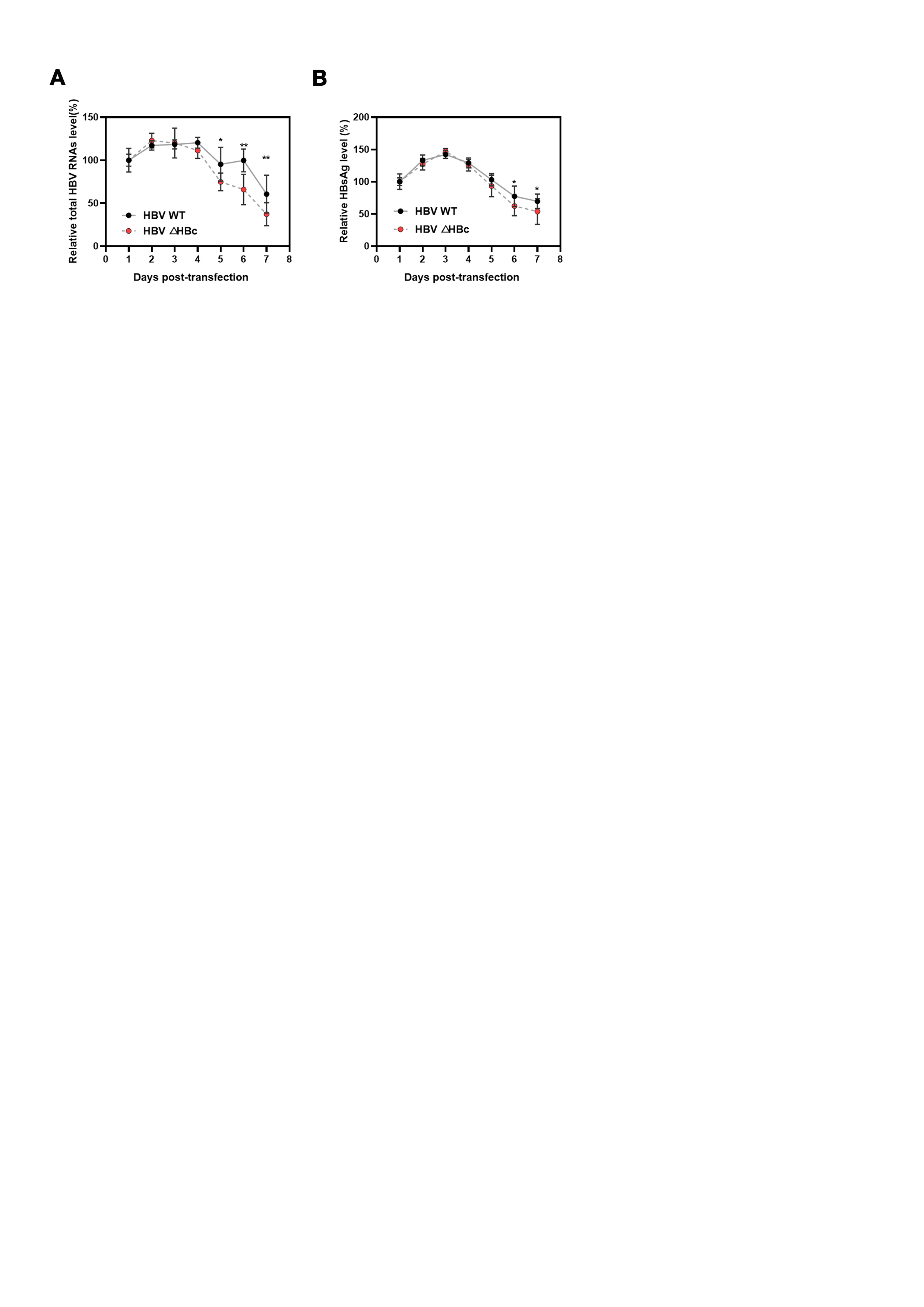

Supplement: Supplementary file 6 — Figure S6 [file 41420_2024_2122_MOESM6_ESM.tif]

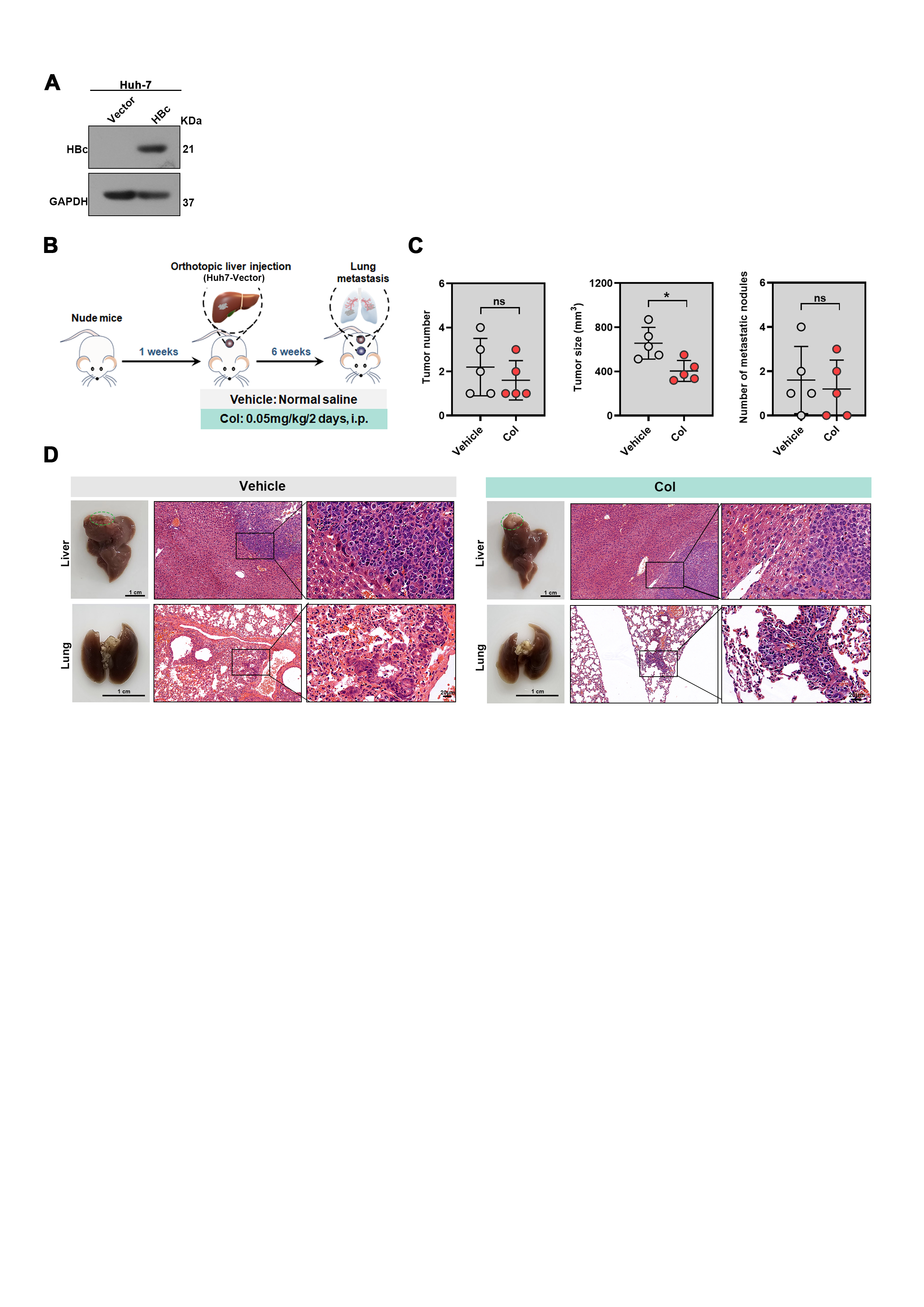

Supplement: Supplementary file 7 — Figure S7 [file 41420_2024_2122_MOESM7_ESM.tif]
