# Supplementary material for: Colchicine-mediated selective autophagic degradation of HBV core proteins inhibits HBV replication and HBV-related hepatocellular carcinoma progression
Source: Cell Death Discov. 2024 Aug 6;10:352. doi: 10.1038/s41420-024-02122-z (PMC11303544; doi:10.1038/s41420-024-02122-z)

Colchicine-mediated selective autophagic degradation of HBV core proteins inhibits HBV replication and HBV-related hepatocellular carcinoma progression

Hui Zhang[1,2], Xiameng Su[2], Leirong Gu[2], Ming Tan[2], Yuting Liu[2], Kexin Xu[2], Jihua Ren[2], Juan Chen[2,3], Zhihong Li[2], Shengtao Cheng[1, 2] *

Table of contents

Supplementary figure legends..2

Supplementary tables..5

Supplementary figure legends

Fig. S1. Screening of colchicine as an inhibitor of the Luc-tagged HBc (Luc-HBc) protein. (A) Huh-7 and HepG2 cells were transfected with a plasmid encoding Luc-HBc for 48 h. The protein level of Luc-HBc was examined by western blotting. (B-E) Huh-7 cells were transfected with a plasmid expressing Luc-HBc and exposed to each compound for 2 days. The expression of Luc-HBc was determined by dual luciferase reporter assay.

Fig. S2. Cell toxicity of colchicine in different cell lines. (A-D) (A) Huh-7 (B), HepG2 (C), HepG2-NTCP and (D) HepAD38 cells were treated with various concentrations of colchicine for 48 h. The effects of colchicine on cell viability were determined by the MTT assay.

Fig. S3. The effects of colchicine on HBV protein and mRNA levels. (A) Huh-7 cells were transfected with plasmids encoding Flag-HBc, the X protein (Flag-HBx), the middle surface protein (Flag-HBs), or polymerase (Flag-HBp). The protein levels of Flag-HBc, Flag-HBx, Flag-HBs and Flag-HBp were analysed by western blotting. (B) Huh-7 cells transfected with Flag-HBc were treated with Col and collected at the specified time for western blotting. (C-F) Huh-7 cells transfected with Flag-HBc, Flag-HBx, Flag-HBs, or Flag-HBp were treated with different

concentrations of Col for 48 h. (C) The mRNA levels of HBc were examined by real-time PCR. The β-actin mRNA level was used as an internal control. (D-F) The protein and mRNA levels of HBx, HBs and HBp were examined. The data are expressed as the mean ± SD of three replicates of the experiment.

Fig. S4. HBc could not bind to p62 directly. (A) Western blot analysis for HBc after GST or GST-p62 pull down. (B) Huh-7 cells were transduced with siCtrl or si p62 and then transfected with a plasmid encoding Flag-HBc. The protein level of Flag-HBc and p62 was examined by western blotting.

Fig. S5. Colchicine inhibited HBV transcription and replication *in vitro*. (A) Mean plasma concentration-time curves of colchicine after IP administration (n=3). (B) HBs levels in liver tissues were analysed by immunohistochemistry. Scale bar = 40 μm.

Fig. S6. The effect of HBc deficiency on HBV RNA and HBsAg. (A-B) Transfect plasmids encoding HBV WT or HBV ΔHBc into Huh-7 cells and samples were collected at indicatied time points. (A) Real-time PCR detection of HBV RNA. (B) Collect cell culture supernatant to detect HBsAg levels. The data are expressed as the mean ± SD of three replicates of the experiment. (*$P < 0.05$; **$P < 0.01$).

Fig. S7. The effect of Colchicine on HCC metastasis in vivo. (A) The overexpression efficiency of HBc in Huh-7 cells was analysed by western blotting. (B) Flow chart showing the method and concentration of colchicine used. The Lung metastasis model was established by orthotopically injecting Huh7-Vector cells (n = 5). (C) Tumor number, tumor size and metastatic nodules were calculated. (D) Representative images of tumor-bearing liver, lung and H&E staining for liver and lung tissues were provided. (*$P < 0.05$).

Supplementary tables

Table S1. Primers, probes and siRNA sequences.

Name	Sequence	Supplier
HBV cccDNA-F	GTGCACTTCGCTTCACCTCT	Beijing Tsingke
HBV cccDNA-R	AGCTTGGAGGCTTGAACAGT	Beijing Tsingke
HBV cccDNA Probe	ACGTCGCATGGAGACCACCG TGAACGCC	Invitrogen
HBV 3.5-kb RNA-F	GCCTTAGAGTCTCCTGAGCA	Beijing Tsingke
HBV 3.5-kb RNA-R	GAGGGAGTTCTTCTTCTAGG	Beijing Tsingke
Total HBV RNA-F	ACCGACCTTGAGGCATACTT	Beijing Tsingke
Total HBV RNA-R	GCCTACAGCCTCCTAGTACA	Beijing Tsingke
β-actin (human)-F	CTCTTCCAGCCTTCCTTCCT	Beijing Tsingke
β-actin (human)-R	AGCACTGTGTTGGCGTACAG	Beijing Tsingke

β-actin (mouse)-F	CCACCATGTACCCAGGCATT	Beijing Tsingke
β-actin (mouse)-R	CGGACTCATCGTACTCCTGC	Beijing Tsingke
HBV DNA-F	CCTAGTAGTCAGTTATGTCAAC	Beijing Tsingke
HBV DNA-R	TCTATAAGCTGGAGGAGTGCGA	Beijing Tsingke
Flag-HBx-F	CAATGTCAACGACCGACCT	Beijing Tsingke
Flag-HBx-R	AGACCAATTTATGCCTACAGCC	Beijing Tsingke
Flag-HBs-F	GCTCAGTTTACTAGTGCCAT	Beijing Tsingke
Flag-HBs-R	CATCAACGCAGGATAACCAC	Beijing Tsingke
Flag-HBc-F	ATGTCAACACTAATATGGGCCTA	Beijing Tsingke
Flag-HBc-R	TCCACACTCCGAAAGACACC	Beijing Tsingke
Flag-HBp-F	GCTCAGTTTACTAGTGCCAT	Beijing Tsingke

Flag-HBp-R	CATCAACGCAGGATAACCAC	Beijing Tsingke
si p62-sense	CGCUCACCGUGAAGGCCUATT	Beijing Tsingke
si p62-antisense	UAGGCCUUCACGGUGAGCGTT	Beijing Tsingke
si ATG5-sense	GACGUUGGUAACUGACAAATT	Beijing Tsingke
si ATG5-antisense	UUUGUCAGUUACCAACGUCTT	Beijing Tsingke
si Ctrl-sense	UUCUCCGAACGUGUCACGU	Beijing Tsingke
si Ctrl-antisense	ACGUGACACGUUCGGAGAA	Beijing Tsingke

Table S2. Antibodies and Reagents

**Name**	**Supplier**	**Cat no.**	**Concentration**
Anti-HBsAg	Novus	NB100-62652	1:1000
Anti-GAPDH	Santa Cruz Biotechnology	sc-47724	1:5000
Anti-LC3B	Cell Signaling Technology	3868S	1:1000
Anti-SQSTM1/p62	Cell Signaling Technology	88588S	1:1000
Anti-Beclin-1	Cell Signaling Technology	3495T	1:1000
Anti-AMPKα	Cell Signaling Technology	5832T	1:1000
Anti-p-AMPKα (Thr172)	Cell Signaling Technology	2535T	1:1000
Anti-ULK1	Cell Signaling Technology	8054T	1:1000
Anti-p-ULK1 (Ser555)	Cell Signaling Technology	5869T	1:1000
Anti-mTOR	Cell Signaling Technology	2983T	1:1000

Anti-p-mTOR (Ser2448)	Cell Signaling Technology	5536T	1:1000
Anti-Ubiquitin	Cell Signaling Technology	3936	1:1000
Anti-ATG5	Proteintech	10181-2-AP	1:1000
Mouse anti-Flag	Sigma–Aldrich	F1804	1:3000
Normal rabbit IgG	Merck Millipore	NI01	1:200
Normal mouse IgG	Merck Millipore	NI03	1:200
GST-Tag	Cell Signaling Technology	2625T	1:3000
His-Tag	Abmart	M30111	1:3000
IPKine™ HRP Goat Anti-Mouse IgG HCS	Abbkine	A25112	1:3000
IPKine HRP AffiniPure Goat Anti-Mouse IgG Light Chain	Abbkine	A25012	1:3000
Anti-HBc	kindly provided Prof. XueFei Cai (Chongqing Medical University, China)		1:1000

78

Name	Supplier	Cat no.
Alkaloid compound	Chengdu Desite Biological Technology Co., Ltd	
MG132	Selleck	S2619
CHX	97064-722	Amresco
3-MA	Selleck	S2767
Chloroquine diphosphate	Selleck	S4157
BafA1	Selleck	S1413
Mitomycin-C	Selleck	S7417

Table S3. Mean numbers of blood cells and parameters for liver and kidney toxicity induced by treatment with colchicine.

Colchicine dose			
	**0 mg/kg**	**0.025 mg/kg**	**0.05 mg/kg**
**Body weight (g)**	21.9±1.13	22.6±0.8	21.2±1.01
**WBC (×10^3/μL)**	10.952±1.241	9.193±1.524	10.024±1.325
**RBC(×10^6/μL)**	3.67±1.72	4.17±0.93	5.13±1.32
**Haemoglobin(g/dL)**	13.5±1.03	12.2±1.68	13.1±1.35
**Platelets(×10^3/μL)**	788±114.86	741±135.86	832±126.76
**Total protein(g/dL)**	6.4±0.27	6.7±0.18	6.2±0.24
**Albumin(g/dL)**	2.7±0.12	2.6±0.09	2.5±0.13
**ALT(U/L)**	57.2±8.47	43.5±11.4	48±13.2
**AST(U/L)**	55.2±11.4	52±15.8	48.7±10.32
**ALP(U/L)**	82±8.15	87±12.46	96±17.84
**GGT(U/L)**	6.8±2.15	7.4±3.02	6.9±2.12
**TBIL (mg/dL)**	1.79±0.73	2.26±1.03	2.06±1.11
**CREA (mg/dL)**	0.422±0.102	0.462±0.032	0.379±0.114
**BUN (mg/dL)**	21.9±2.31	22.8±3.41	20.38±2.38

The data are expressed as the means ± standard deviations.

Abbreviations: WBC, white blood cell; RBC, red blood cell;

ALT, alanine transaminase; AST, aspartate transaminase; ALP, alkaline phosphatase; GGT, γ-glutamyl transpeptidase; TBIL, total bilirubin; CREA, creatinine; BUN, blood urea nitrogen. ns, no significant difference ($p > 0.05$) compared with the different groups.

Supplementary Material 2

Original western blots

Figure 1

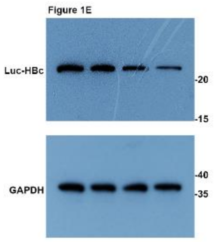

Figure 2

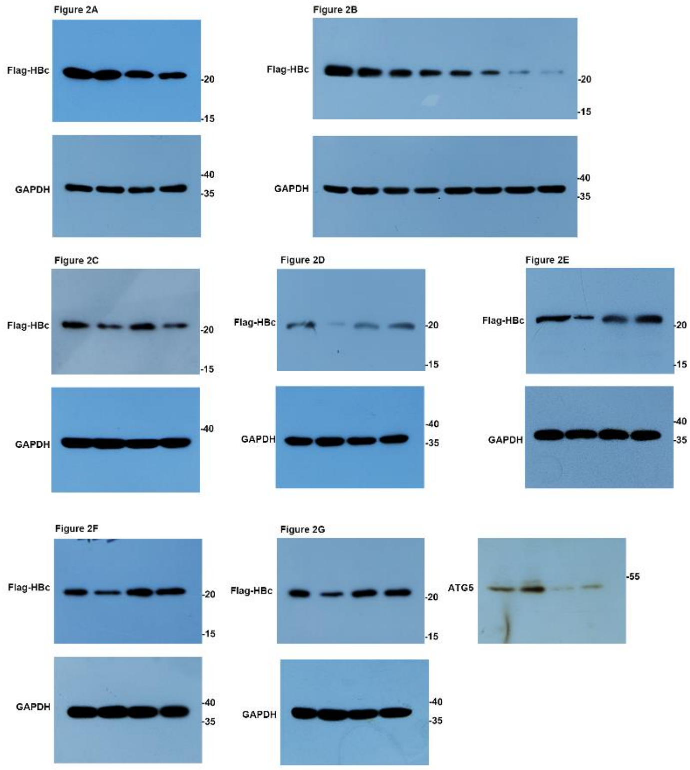
Figure 2A
Flag-HBc
-20
-15
GAPDH
-40
-35
Figure 2B
Flag-HBc
-20
-15
GAPDH
-40
-35
Figure 2C
Flag-HBc
-20
-15
GAPDH
-40
Figure 2D
Flag-HBc
-20
-15
GAPDH
-40
-35
Figure 2E
Flag-HBc
-20
-15
GAPDH
-40
-35
Figure 2F
Flag-HBc
-20
-15
GAPDH
-40
-35
Figure 2G
Flag-HBc
-20
-15
GAPDH
-40
-35
ATG5
-55

Figure 3

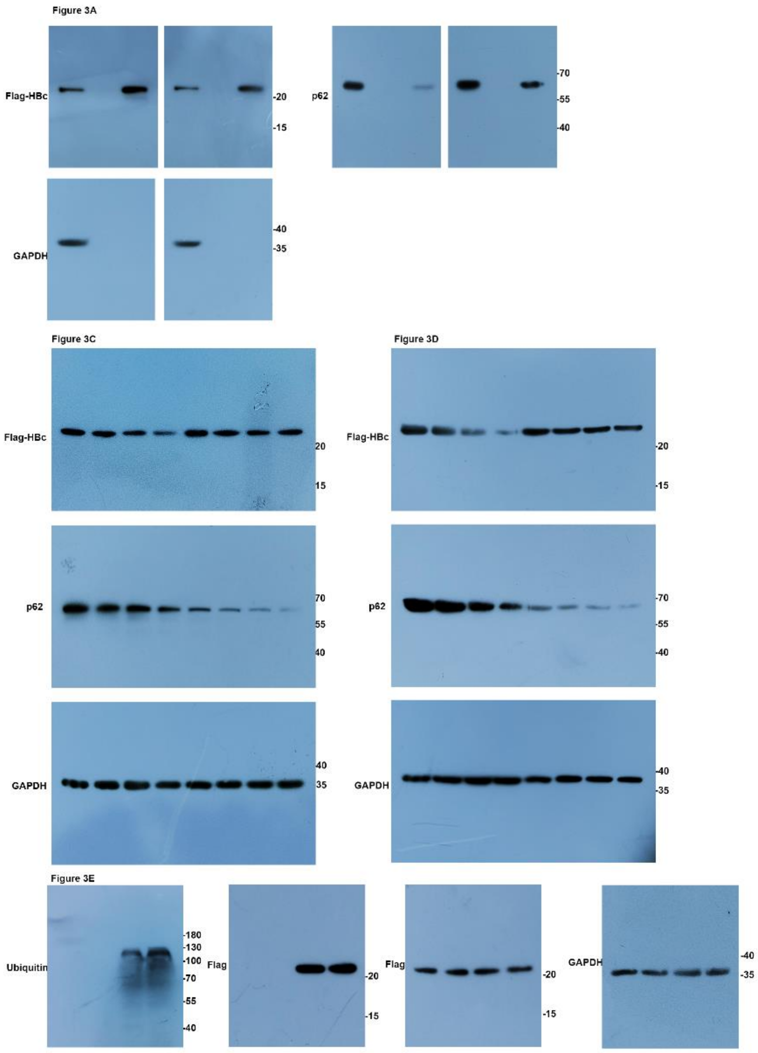
Figure 3A
Flag-HBc
-20
-15
p62
-70
-55
-40
GAPDH
-40
-35
Figure 3C
Figure 3D
Flag-HBc
20
15
p62
70
55
40
GAPDH
40
35
Figure 3E
Ubiquitin
-180
-130
-100
-70
-55
-40
Flag
-20
-15
GAPDH
-40
-35

Figure 4

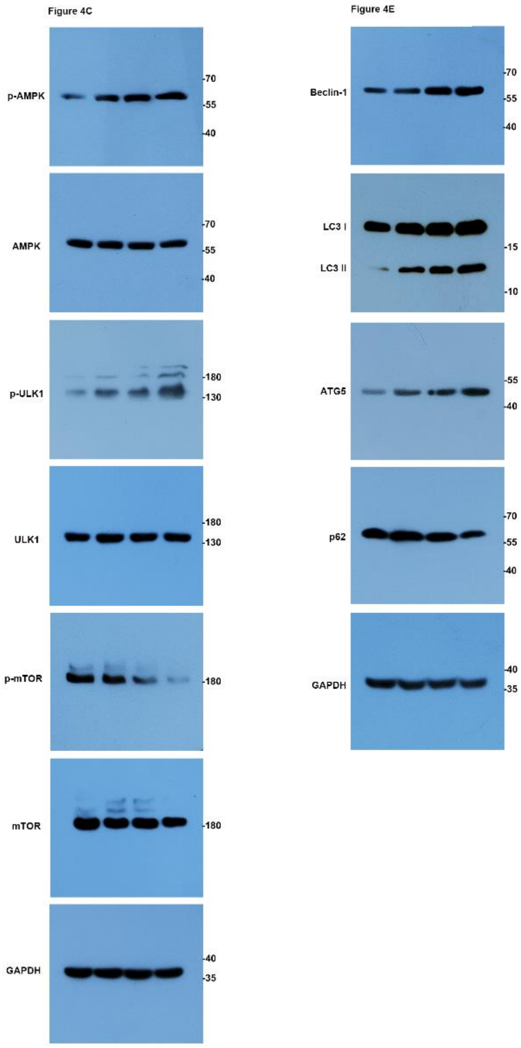
Figure 4C
p-AMPK
AMPK
p-ULK1
ULK1
p-mTOR
mTOR
GAPDH
70
55
40
180
130
35
Figure 4E
Beclin-1
LC3 I
LC3 II
ATG5
p62
GAPDH
15
10

Figure 5

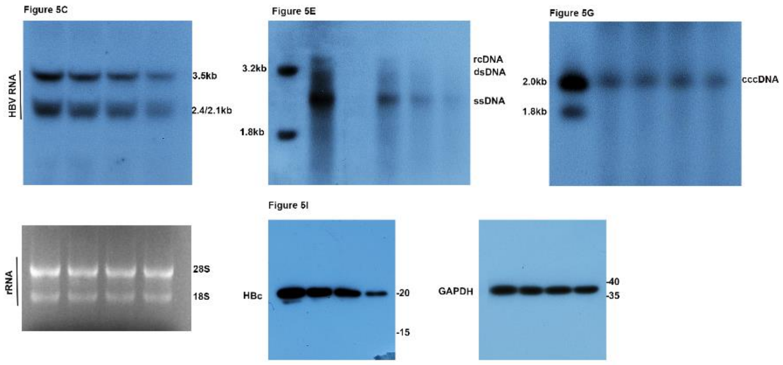
Figure 5C
HBV RNA
3.5kb
2.4/2.1kb
rRNA
28S
18S
Figure 5E
3.2kb
1.8kb
rcDNA
dsDNA
ssDNA
Figure 5G
2.0kb
1.8kb
cccDNA
Figure 5I
HBc
-20
-15
GAPDH
-40
-35

Figure 6

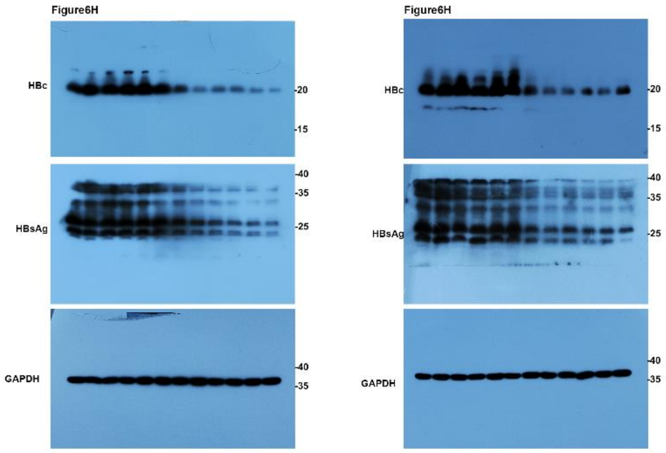
Figure6H
HBc
-20
-15
HBsAg
-40
-35
-25
GAPDH
-40
-35
Figure6H
HBc
-20
-15
HBsAg
-40
-35
-25
GAPDH
-40
-35

Figure 7

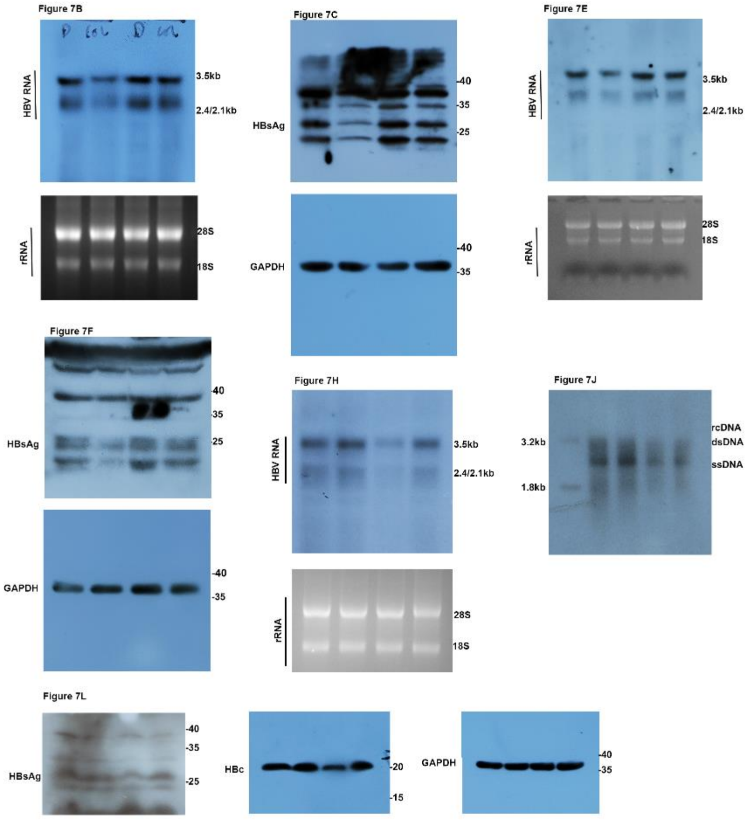

Figure 7B
HBV RNA
3.5kb
2.4/2.1kb
rRNA
28S
18S
Figure 7C
HBsAg
-40
-35
-25
GAPDH
-40
-35
Figure 7E
HBV RNA
3.5kb
2.4/2.1kb
rRNA
28S
18S
Figure 7F
HBsAg
40
35
25
GAPDH
-40
-35
Figure 7H
HBV RNA
3.5kb
2.4/2.1kb
rRNA
28S
18S
Figure 7J
3.2kb
1.8kb
rcDNA
dsDNA
ssDNA
Figure 7L
HBsAg
-40
-35
-25
HBc
-20
-15
GAPDH
-40
-35

Supplementary Fig. 1

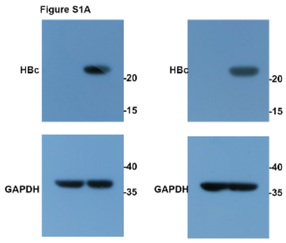

Supplementary Fig. 3

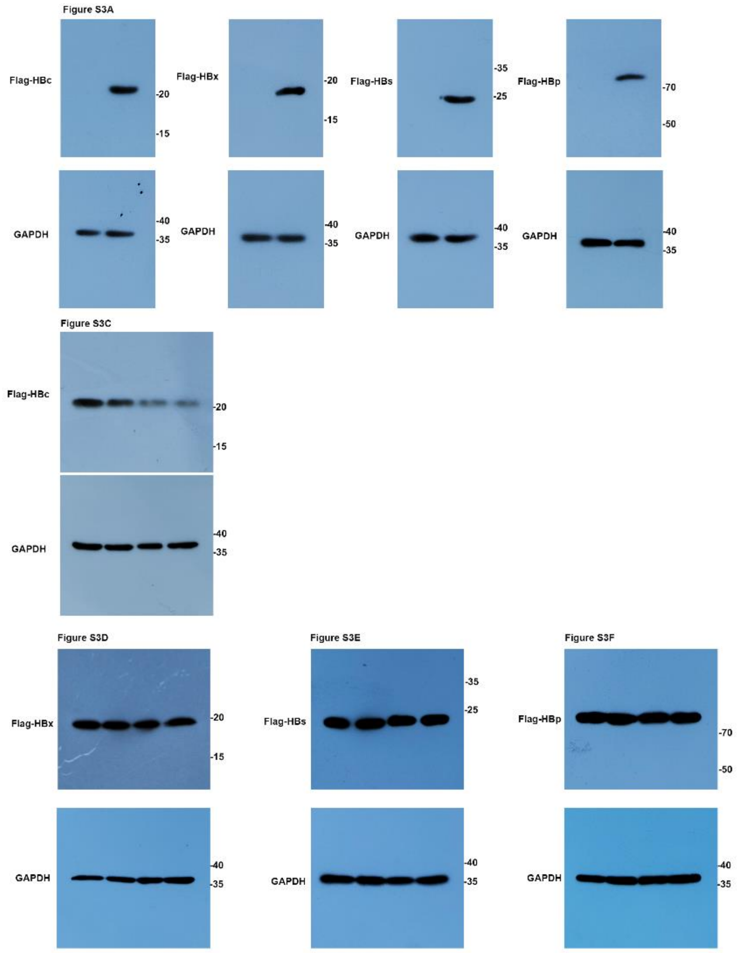

Supplementary Fig. 4

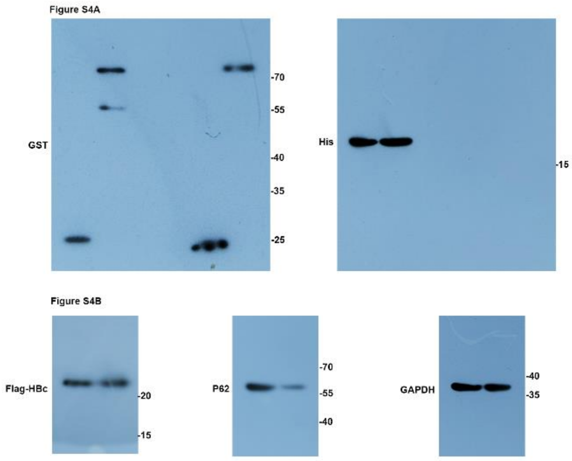

Supplementary Fig. 7

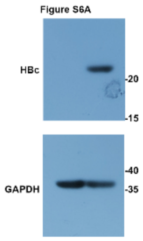

Supplement: Supplementary file 8 — Supplemental Material [file 41420_2024_2122_MOESM8_ESM.pdf]
